# RHOX10 drives mouse spermatogonial stem cell establishment through a transcription factor signaling cascade

**DOI:** 10.1016/j.celrep.2021.109423

**Published:** 2021-07-20

**Authors:** Kun Tan, Hye-Won Song, Miles F. Wilkinson

**Affiliations:** 1 Department of Obstetrics, Gynecology, and Reproductive Sciences, School of Medicine, University of California, San Diego, La Jolla, CA 92093, USA; 2 Institute of Genomic Medicine, University of California, San Diego, La Jolla, CA 92093, USA; 3 Lead contact

## Abstract

Spermatogonial stem cells (SSCs) are essential for male fertility. Here, we report that mouse SSC generation is driven by a transcription factor (TF) cascade controlled by the homeobox protein, RHOX10, which acts by driving the differentiation of SSC precursors called pro-spermatogonia (ProSG). We identify genes regulated by RHOX10 in ProSG *in vivo* and define direct RHOX10-target genes using several approaches, including a rapid temporal induction assay: iSLAMseq. Together, these approaches identify temporal waves of RHOX10 direct targets, as well as RHOX10 secondary-target genes. Many of the RHOX10-regulated genes encode proteins with known roles in SSCs. Using an *in vitro* ProSG differentiation assay, we find that RHOX10 promotes mouse ProSG differentiation through a conserved transcriptional cascade involving the key germ-cell TFs DMRT1 and ZBTB16. Our study gives important insights into germ cell development and provides a blueprint for how to define TF cascades.

## In brief

Using a battery of genome-wide approaches, Tan et al. identify genes regulated by the RHOX10 transcription factor in mouse pro-spermatogonia. Temporal waves of RHOX10 targets, including both direct and indirect targets, are defined. Among these genes are those that participate in a conserved transcription factor signaling cascade driving pro-spermatogonia differentiation.

## Graphical abstract

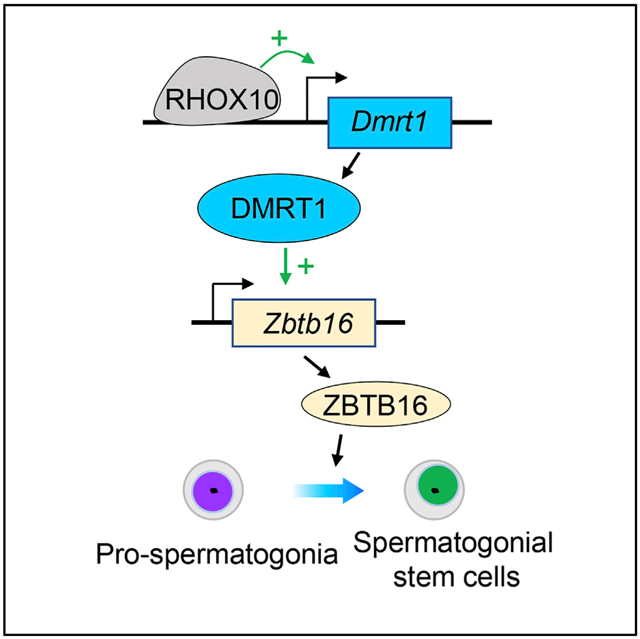

## INTRODUCTION

The propagation of the male germline depends on a specialized cell type called the spermatogonial stem cell (SSC). Like all stem cells, SSCs have the capacity to both self-renew and differentiate (Kubota and Brinster, 2018; Tan and Wilkinson, 2019). Indeed, SSCs have unlimited self-renewal capacity and thus are essential to maintain spermatogenesis into late adulthood. When induced to differentiate, SSCs give rise to a series of germ cell stages that ultimately generate sperm. There is considerable interest in SSCs as a system to understand stem cell biology and as a therapeutic target to cure male infertility (Kubota and Brinster, 2018; Tan and Wilkinson, 2020).

SSCs are derived from precursor germ cells called pro-spermatogonia (ProSG; otherwise known as “gonocytes”). Mouse ProSG are composed of at least three stages, based on morphology and proliferative status: multiplying (M)-ProSG, primary transitional (T1)-ProSG, and secondary transitional (T2)-ProSG (Culty, 2013; McCarrey, 2013). M-ProSG are proliferative cells that form from primordial germ cells (PGCs) in fetal mice. M-ProSG give rise to mitotically quiescent T1-ProSG, which undergo genome-wide *de novo* DNA methylation to replace the DNA methylation marks erased at the PGC stage (Yamanaka et al., 2019). This epigenomic reprogramming continues after T1-ProSG convert into T2-ProSG at ~postnatal day-2 (P2). T2-ProSG re-initiate proliferation and are regarded as the direct precursors of SSCs (Culty, 2013; McCarrey, 2013). Single-cell RNA sequencing (scRNA-seq) analysis has provided new insights into the ProSG-to-SSC transition. For example, Law et al. used scRNA-seq analysis to identify a cell cluster that likely correspond to the T2-ProSG subset that give rise to SSCs (Law et al., 2019). Using germ-cell transplantation analysis, they obtained evidence that ProSG are fated to become SSCs at the fetal stage (Law et al., 2019). Tan et al. used scRNA-seq analysis to identify discrete ProSG cell clusters from embryonic day (E) 18.5 and P2 testes that largely correspond to the T1- and T2-ProSG, respectively (Tan et al., 2020c). This study also identified gene and protein markers for ProSG subsets and emergent SSCs that are potentially valuable resources for the field.

The molecular mechanisms by which ProSG transition to form SSCs is largely unknown. While several proteins have been shown to play roles in SSC establishment, their exact roles have remained unclear (Fok et al., 2017; Kang et al., 2016; Xu et al., 2015). We previously reported that a member of the X-linked *Rhox* homeobox gene cluster drives mouse SSC establishment (Song et al., 2016). Loss of *Rhox10* causes progressive loss of spermatogenesis, leading to aberrant germ cell populations in seminiferous tubules, including tubules completely devoid of germ cells, a defect phenocopied by mice lacking the entire *Rhox* cluster (Song et al., 2016). Using a battery of approaches, including scRNA-seq and germ-cell transplantation analyses, it was found that *Rhox10*-null mice abnormally accumulate ProSG and generate few SSCs during the perinatal stage when these stem cells initially form. These data suggest that *Rhox10* acts in ProSG to promote their differentiation into SSCs.

In this communication, we report molecular mechanisms by which RHOX10 acts in mouse germ cells. Using both *in vivo* and *in vitro* genome-wide assays, we identify genes regulated and targeted by RHOX10 in ProSG. We define temporal waves of genes responding to RHOX10. Using rescue and mimic experiments, we identify a TF cascade that acts downstream of RHOX10 to drive ProSG differentiation and SSC establishment.

## RESULTS

### Identification of RHOX10-regulated genes in the male germline *in vivo*

**Figure 1.**
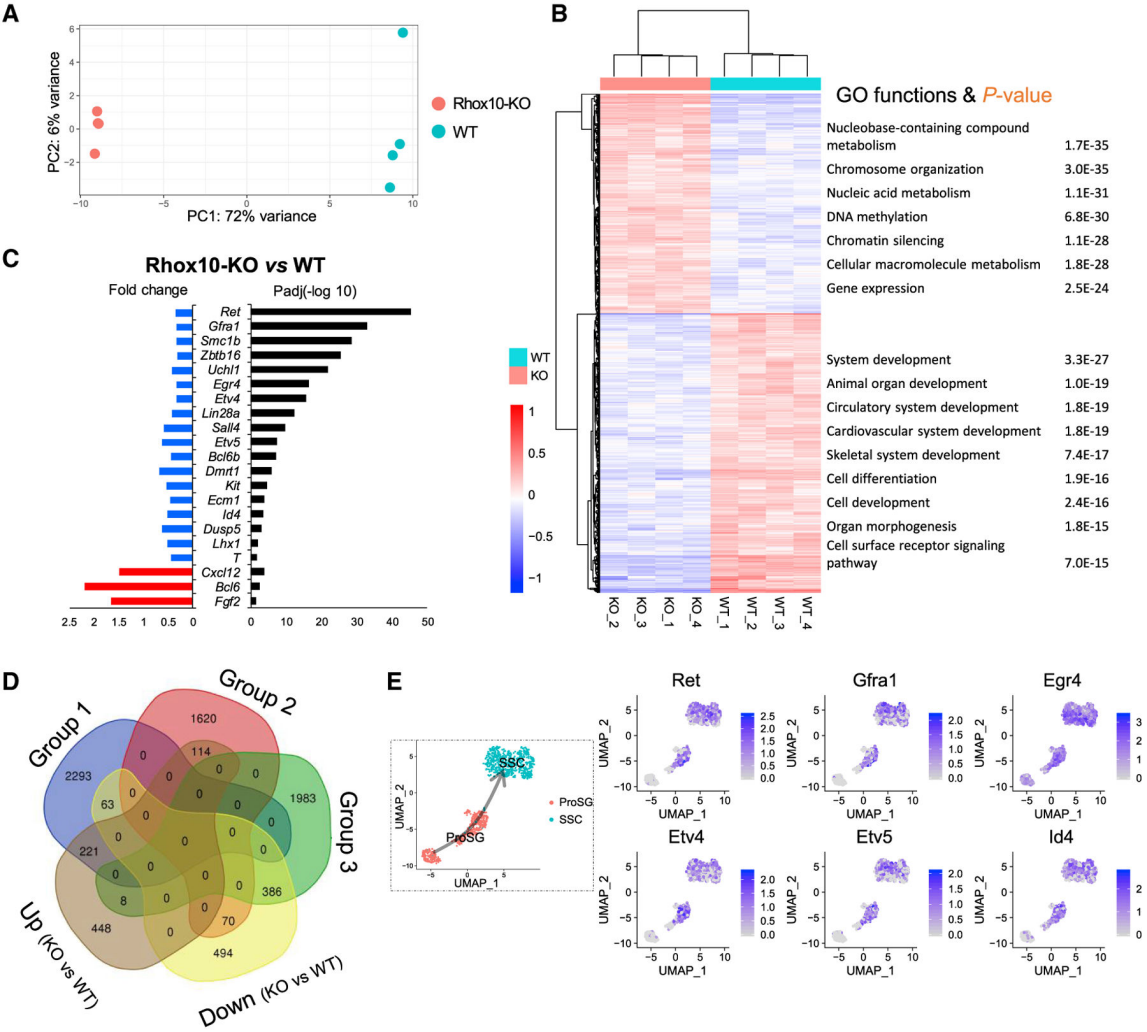
Identification of RHOX10-regulated genes in developing germ cells *in vivo* (A) PCA of RNA-seq datasets from purified germ cells from *Rhox10*-null (Rhox10-KO) and control (WT) P1 testes (the 4 biological replicates we generated from each genotype are shown). (B) Left, hierarchical clustering of DEGs defined by RNA-seq analysis from the samples shown in (A). Right, representative biological processes enriched in upregulated and downregulated DEGs (statistical significance shown). (C) Representative DEGs shown in (B), with a focus on genes with known male germline functions. Left, fold change (*Rhox10*-null versus control). Blue, downregulated genes; red, upregulated genes. Right, false discovery rate (FDR)-adjusted p values determined using DESeq2 (−log10-transformed). (D) DEGs overlapping between the scRNA-seq (Figure S1C) and RNA-seq (B) datasets. (E) Left, UMAP plot showing the annotated ProSG and emergent SSC populations during the perinatal stage, as determined by scRNA-seq analysis of germ cells from E18.5, P2, and P7 mouse testes (Tan et al., 2020c). Right, UMAP plots showing expression of the indicated marker genes. See also Figure S1 and Table S1.

As a first step toward identifying RHOX10-regulated genes potentially important for ProSG differentiation and SSC generation, we performed RNA sequencing (RNA-seq) analysis on *Rhox10*-null versus control germ cells. Given that germ cells from P1 mice are largely pure ProSG, we used P1 Oct4-eGFP^+^ germ cells purified by fluorescence-activated cell sorting (FACS) for RNA-seq analysis. Principal component analysis (PCA) showed that the four *Rhox10*-null ProSG samples clustered together, as did the four control samples, indicative of reproducible results from a given genotype (Figure 1A). In contrast, the four *Rhox10*-null and four control samples plotted distant from each other (Figure 1A), indicating that loss of *Rhox10* causes a major alteration in the ProSG transcriptome. Indeed, 1,013 genes were downregulated and 794 genes were upregulated in *Rhox10*-null germ cells as compared with control germ cells (q < 0.01, ∣log 2 FoldChange∣ >0.5; Figure 1B; Table S1). Among these differentially expressed genes (DEGs) are 93 TF genes (of 813 TF genes expressed in P1 germ cells [11%]; Figure S1A; Kanamori et al., 2004; Zhou et al., 2017). This raises the possibility that RHOX10 controls TF networks driving ProSG-to-SSC differentiation, a possibility we address below. Among these *Rhox10*-regulated TF genes are several known to play roles in SSCs (including *Bcl6b*, *Dmrt1*, *Egr4*, *Etv4*, *Lhx1*, *Pou3f1*, *Sall4*, *T*, and *Usf1*), embryonic stem cells (*Taf5l*, *Klf2*, *Satb2*, and *Hes1*), hematopoietic stem cells (*Cbx2*, *Dnmt1*, *Hhex*, *Irf2*, *Gata2*, *Meis1*, *Nfkb2*, and *Mybl2*), and other stem cells (*Plagl1*, *Notch3*, *Gli2*, *Nr2f2*, *Sox11*, *Hic1*, *Tcf4*, and *Lhx2*). Many non-TFs genes regulated by *Rhox10* are also known to have functions in SSCs or serve as SSC markers (Figure 1C). Gene Ontology (GO) analysis showed that genes negatively regulated by *Rhox10* are enriched for functions associated with nucleic acid metabolism and transcriptional regulation (Figure 1B), which underscores the potential role of *Rhox10* in TF networks. *Rhox10* positively regulated genes are enriched for developmental functions (Figure 1B), consistent with the evidence that *Rhox10* drives ProSG differentiation (Song et al., 2016).

To further assess the nature of these *Rhox10*-regulated genes, we evaluated their expression pattern in germ cells present during the approximate time period when *Rhox10* acts *in vivo* (Song et al., 2016). To achieve this, we examined the expression pattern of these genes in germ cell subsets defined by scRNA-seq analysis independently performed on dissociated cells from freshly isolated whole testes obtained from embryonic day (E) 18.5, P2, and P7 mice (Tan et al., 2020c). Using Monocle pseudotime analysis, we aligned perinatal testicular cells according to stages (Tan et al., 2020c) and observed a clear ProSG-to-SSC trajectory (Figure S1B). We then identified groups of genes that exhibited similar temporal expression patterns along this trajectory (Figure S1C). This revealed that most differentially expressed genes fell into 3 groups: those with peak expression in ProSG (group 1), emergent SSCs (group 3), or at an intermediate stage (group 2) (Figure S1C). Comparison with *Rhox10*-regulated genes revealed that genes downregulated by *Rhox10* overlapped most with group 1 genes, while genes upregulated by *Rhox10* overlapped most with group 3 genes (Figure 1D). These data suggested that *Rhox10* primarily promotes the expression of SSC genes and mainly inhibits the expression of ProSG genes. This regulatory pattern is consistent with RHOX10 promoting ProSG differentiation and SSC generation.

Among the group 1 genes positively regulated by *Rhox10* (downregulated in *Rhox10*-null cells) are genes with well-established functions in SSCs that initiate their expression late during ProSG development and sustain their expression in SSCs; e.g., *Egr4*, *Etv4*, *Etv5*, *Gfra1*, *Id4*, and *Ret* (Figure 1E). The finding that these pro-SSC genes depend on *Rhox10* for maximal expression in the late ProSG-to-SSC stage is intriguing, as it raises the possibility that RHOX10 acts as a central TF controlling many genes for this key stage of germ cell development.

### Identification of genome-wide RHOX10-occupancy sites

To identify candidate RHOX10-direct targets, we used a highly sensitive method that was recently developed to identify TF occupancy sites: cleavage under targets and tagmentation (CUT&Tag) analysis (Kaya-Okur et al., 2019). We performed CUT&Tag analysis on germline stem (GS) cells infected with a lentiviral vector expressing HA-tagged *Rhox10* at a level similar to endogenous *Rhox10* (Figure S2A). To avoid the confounding effects of endogenous *Rhox10*, we knocked it down ~85% by transducing a *Rhox10* small hairpin RNA (shRNA) lentivirus (Figure S2A) and engineered HA-tagged *Rhox10* to be insensitive to this shRNA. Reporter analysis with RHOX10 target genes demonstrated that the introduction of the HA tag at the C terminus of RHOX10 does not interfere with RHOX10’s TF function (Figure S2B).

**Figure 2.**
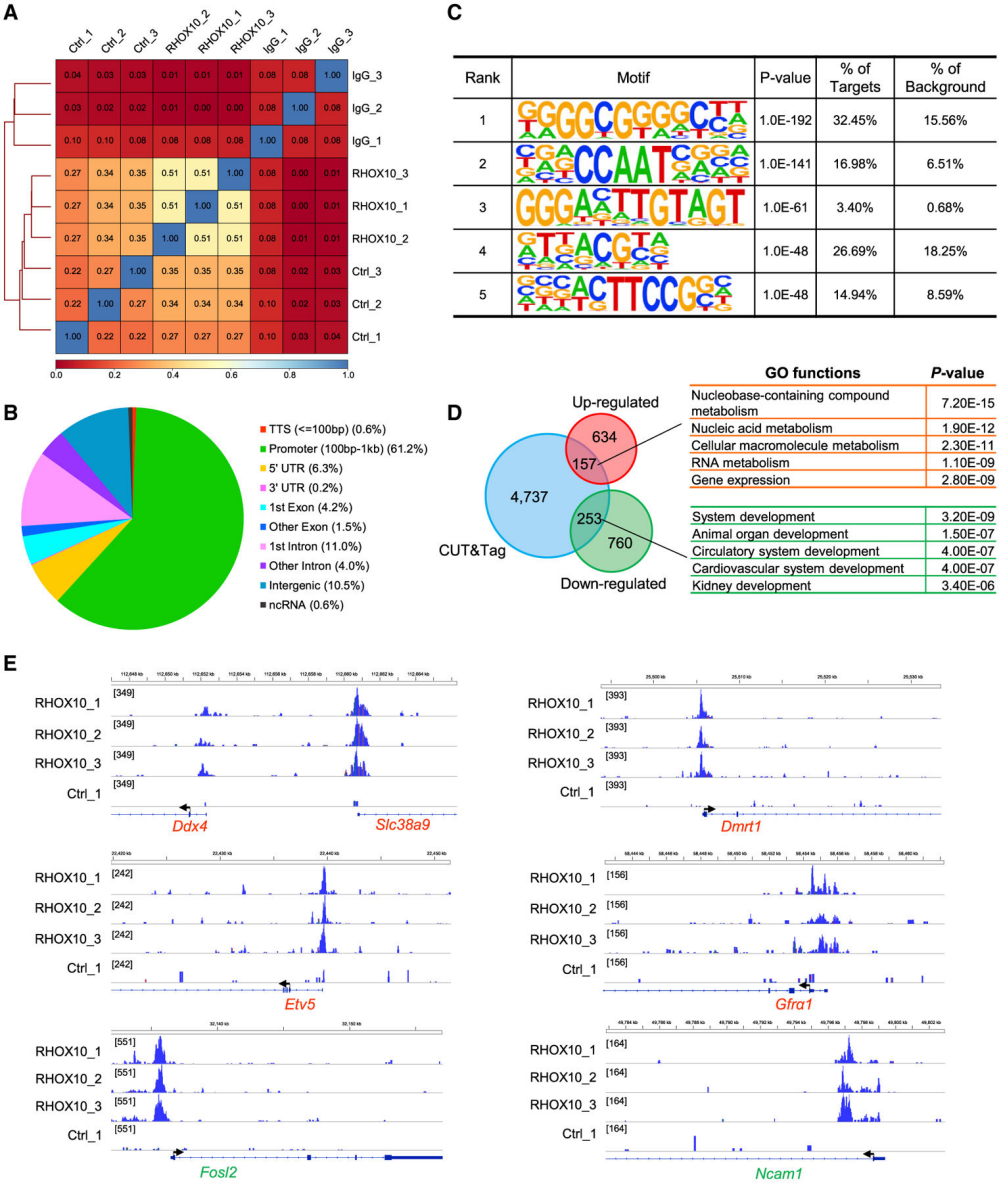
Identification of RHOX10-occupancy sites (A) Pearson correlation analysis of the indicated GS cell samples (performed in triplicate; both IgG and Ctrl are negative controls, as described in the STAR Methods). (B) Location of RHOX10-occupancy peaks relative to the nearest annotated gene. (C) Sequence motifs enriched at RHOX10-occupancy sites, as determined using HOMER. (D) Left, overlapping genes identified by CUT&Tag and RNA-seq analyses. Right, representative biological processes enriched among the overlapping genes. (E) Integrative genomics viewer images showing tracks of normalized RHOX10-occupancy tag counts. Shown are representative overlapping genes identified in (D). Red, *Rhox10*-null downregulated genes bound by RHOX10; green, *Rhox10*-null upregulated genes bound by RHOX10. The arrow denotes the TSS. The 3 biological replicates performed are shown, along with the negative control (Ctrl) described in (A). See also Figure S2 and Table S1.

We performed three RHOX10 CUT&Tag replicates (Figure 2A). Analysis of the three replicates revealed 5,147 annotated peaks (RHOX10-occupancy sites) that were statistically enriched (Table S1). A large proportion of RHOX10-occupancy sites (61.2%) are located in the promoter region, including some occupancy sites very near the transcription start site (TSS) (Figures 2B and S2C). This remarkable enrichment strongly suggests that a major function of RHOX10 is to regulate the expression of protein-coding genes. The most statistically enriched sequence motifs in RHOX10-occupany sites are listed in Figure 2C. These putative RHOX10-binding sites are similar with those of other TFs, including KLF5, BCL6B, ASCL2, NFYA, ETV5, and ETV6 (Figure S2D), all of which are known to have roles in SSCs and other stem cells (Bungartz et al., 2012; Parisi et al., 2008; Song and Wilkinson, 2014; van der Flier et al., 2009). This suggests that RHOX10 has the potential to regulate some of the same downstream genes as these other TFs, albeit in different biological contexts.

To identify candidate RHOX10-target genes, we examined the overlap of RHOX10 CUT&Tag peaks (Table S1) with *Rhox10*-null regulated genes defined by RNA-seq analysis (Figure 1B; Table S1). This revealed 410 genes that are both regulated and occupied by RHOX10, with 157 and 253 genes up- and downregulated, respectively, in *Rhox10*-null germ cells (Figure 2D). GO analysis showed that the upregulated genes are associated with several functions, including “metabolism” and “gene expression,” while the downregulated genes are mainly associated with “development” (Figure 2D). Among these candidate RHOX10-direct target genes are *Ddx4*, *Dmrt1*, *Etv5*, *Gfrα1*, *Fosl2*, and *Ncam1*, all of which are known to have roles in germ cells or stem cells (Figure 2E). Interestingly, 31 of these candidate RHOX10-direct target genes encode TFs (Table S1). 18 of these TFs are downregulated after *Rhox10* depletion and thus are presumably positively regulated by *Rhox10*. Among them, *Bcl6b*, *Dmrt1*, *Egr4*, *Pou3f1*, *Sall4*, and *T* have been reported to have roles in SSCs (Hobbs et al., 2012; Hogarth et al., 2010; Oatley et al., 2006; Wu et al., 2010, 2011; Zhang et al., 2016). Among the 13 TFs upregulated by *Rhox10* loss are *Irf2*, *Plagl1*, and *Gli2*, all of which have been reported to have roles in other stem cells (Minamide et al., 2020; Schmidt-Edelkraut et al., 2013; Zhao et al., 2017). The finding that RHOX10 is likely to directly regulate many TFs involved in stem cells raises the possibility that RHOX10 drives SSC establishment via a TF cascade, which we test below.

### Identification of RHOX10-induced gene waves

**Figure 3.**
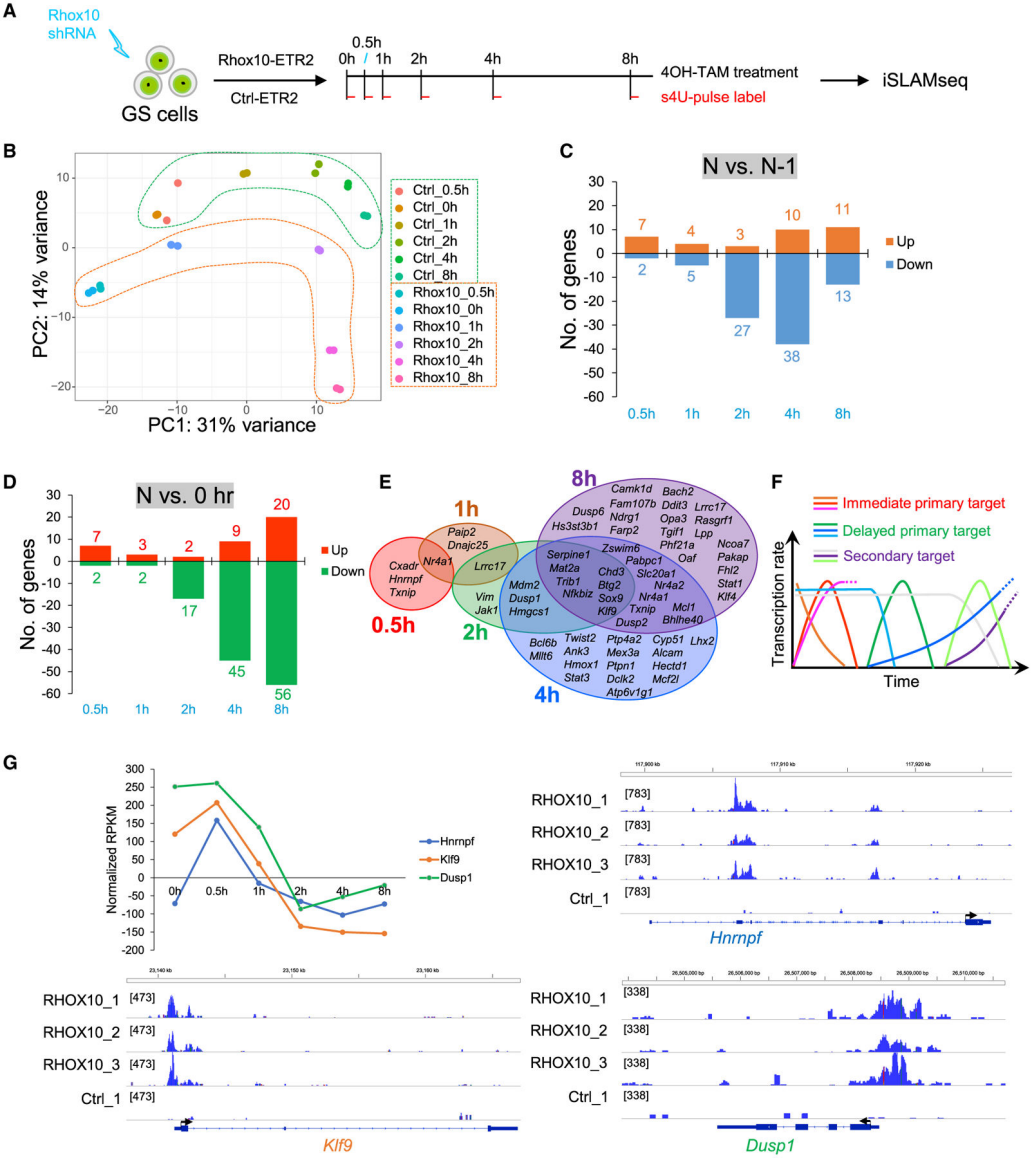
Gene waves induced by RHOX10 (A) Schematic illustration of iSLAMseq workflow. Both *Rhox10*-ERT2 and Control-ERT2 transduced cells were incubated with 4OH-TAM for 0, 0.5, 1, 2, 4, and 8 h, respectively. Before collecting samples, cells were given a pulse of s^4^U for 15 min. (B) PCA of iSLAMseq samples. (C) DEGs between each time point (e.g., 0.5 h compared with 0 h; 1 h compared with 0.5 h). (D) DEGs at each time point compared with 0 h time point. (E) RHOX10-regulated genes (as measured using iSLAMseq) bound by RHOX10 (as measured by CUT&Tag) exhibiting sustained regulation at multiple time points (as determined by the method in either C or D). (F) Gene classes transcriptionally responding to RHOX10. Immediate and delayed primary targets are genes directly regulated by RHOX10, with rapid or slow kinetics, respectively. Both positively and negatively regulated genes are shown. Secondary targets are regulated by RHOX10 through an intermediary and thus have delayed kinetics. For easier visualization, the different classes of genes are shown with slightly different slopes, even though the actual slope will vary depending on the specific gene, not the gene class. (G) Top left, normalized RPKM reads reflecting relative transcription rates of the genes shown after 4OH-TAM treatment. Bottom and top right, integrative genomics viewer images showing tracks of normalized tag counts of RHOX10 occupancy, as measured by CUT&Tag. The 3 biological replicates performed on GS cells expressing HA-tagged RHOX10 are shown. The negative control (Ctrl) is lysates from GS cells transduced with a HA-tagged empty vector. See also Figure S3 and Table S2.

TF occupancy within or near a gene promoter or enhancer region is often assumed to be sufficient evidence for the gene to be classified as a direct TF target (MacQuarrie et al., 2011). This is not valid, as TFs residing within a gene promoter or enhancer will not necessarily regulate the transcription of the associated gene. Thus, TF occupancy assays (e.g., chromatin immunoprecipitation sequencing [ChIP-seq] or, in our case, CUT&Tag) are not sufficient to define function. To more reliably identify direct TF targets, we designed a rapid induction assay–inducible-thiol(SH)-linked alkylation for the metabolic sequencing of RNA (iSLAMseq)–which detects newly synthesized transcripts–genome-wide–in response to a TF. In this assay, transcription is measured using an orthogonal chemistry approach that measures the nucleotide analog, 4-thiouridine (s^4^U), by reverse-transcription-dependent thymine-to-cytosine (T > C) conversions by high-throughput sequencing (Herzog et al., 2017). For precise temporal analysis, the TF of interest is modified so it can be activated in an expeditious manner (Figure 3A).

We performed iSLAMseq analysis in GS cells derived from neonatal (P0-P2) male testes (Kanatsu-Shinohara et al., 2005), a developmental window just before the conversion of ProSG to SSCs (see Introduction). To rapidly induce RHOX10, we chose to employ the estrogen receptor T2 (ERT2) system, which drives swift translocation of the TF of interest to the nucleus in response to 4-hydroxytamoxifen (4OH-TAM) (Matsuda and Cepko, 2007). Thus, we generated a construct encoding a RHOX10-ERT2 fusion protein (Figure S3A) and transduced it, along with a *Rhox10*-shRNA lentiviral construct to knockdown endogenous RHOX10, into GS cells. As a negative control, we transduced GS cells with both a *Rhox10*-shRNA lentiviral construct and an empty ERT2 construct. As per the standard SLAMseq protocol (Herzog et al., 2017), the labeled RNAs was alkylated via iodoacetamide (IAA) treatment, which converts thymine (T) to cytosine (C). As expected, we found the main conversion is T to C (Figure S3B). Comparison with the negative control (–IAA) revealed that s^4^U is efficiently incorporated into the samples (~1.5%–3.5% versus <0.15% background incorporation; Figure S3B). PCA showed that the 2 replicates for each time point (0, 0.5, 1, 2, 4, and 8 h) were closely aligned, while samples from different time points were well separated (Figure 3B), indicating that the transcriptional profile changed significantly at the different time points tested.

Analysis of the iSLAMseq datasets revealed many genes exhibiting shifts in transcriptional activity at different time points after RHOX10 activation (Table S2). Figure 3C shows genes exhibiting statistically significant transcription rate changes between time points, while Figure 3D shows genes undergoing statistically significant transcription rate changes compared to the 0 h time point. Among these genes with different kinetics of their transcriptional responsiveness to RHOX10 are a large number of genes encoding TFs and DNA-binding proteins (*Twist2*, *Bcl6b*, *Dot1l*, *Egr2*, *Nr4a2*, *Cebpd*, *Egr3*, *Stat1*, *Sox9*, *Klf4*, *Egr1*, *Junb*, *Ddit3*, *Jun*, *Fus*, *Fhl2*, *Mllt6*, and *Nr4a1*) (Figure 3E). Some of these TFs, including *Bcl6b*, *Sox9*, and *Junb*, have been reported to play roles in SSCs, although their roles in ProSG differentiation are unknown. *Bcl6b and Ddit3* are downregulated and upregulated, respectively, in *Rhox10*-null P1 germ cells, as shown by RNA-seq (Table S1), while *Twist2*, *Bcl6b*, *Nr4a2*, *Stat1*, *Sox9*, *Klf4*, *Ddit3*, *Fhl2*, *Mllt6*, and *Nr4a1* are bound by RHOX10, as detected by CUT&Tag (Table S1). This raises the possibility that RHOX10 regulates many genes via these TFs, a notion further supported by the finding that “transcription” is an enriched GO category of genes transcriptionally regulated by RHOX10 (Table S2). RHOX10 also transcriptionally regulates many genes known to play important roles in germ cell development (e.g., *Sox9*, *Bcl6b*, *Junb*, *Kdm6b*, and *Inhba*), and those with known roles in SSCs and other stem cells (e.g., *Pim1*, *Id1*, *Egr1*, *Dot1l*, *Cdkn1a*, *Klf9*, and *Klf4*). GO analysis revealed that iSLAM-seq-defined RHOX10-regulated genes are associated with functions including “regulation of cell proliferation,” “response to mechanical stimulus,” and “negative regulation of apoptosis” (Table S2). KEGG pathway analysis showed that these RHOX10-regulated genes are enriched for proteins involved in “TNF signaling,” “p53 signaling,” “oxytocin signaling,” “thyroid hormone signaling,” and “FoxO signaling” (Table S2).

Cross-analysis with the CUT&Tag dataset indicated that approximately 1/3 to 1/2 of RHOX10-regulated genes at each time point contained statistically significant RHOX10 occupancy peaks (Table S2; 44%, 31%, 36%, 42%, and 40% of the RHOX10-regulated genes exhibiting statistically significant altered transcription rates at 0.5, 1, 2, 4, and 8 h, respectively, were detectably occupied by RHOX10). This suggests that a relatively high proportion of RHOX10-regulated genes, even those with delayed kinetics, are direct targets.

We classified RHOX10-regulated genes as “immediate-early RHOX10-direct targets” if they (1) were bound by RHOX10 (as determined by CUT&Tag) and (2) exhibited significantly altered transcription rates after only 0.5 h and/or 1 h exposure to RHOX10-ERT2 and 4OH-TAM (Figures 3E and 3F). This class includes genes encoding the TF NR4A1 and RNA-binding proteins involved in RNA splicing (HNRNPF) or translation (PAIP2). Interestingly, some of the immediate-early direct targets exhibit transient regulation (Figures 3E and 3F). For example, *Hnrnpf* is transcriptionally induced and then rapidly transcriptionally extinguished within 1 h of 4OH-TAM treatment (Figure 3G). Some exhibit rapid transcriptional shut-off, e.g., *Klf9* and *Dusp1* (Figure 3G). A second subclass of these immediate early targets exhibit sustained transcriptional alterations in response to RHOX10, e.g., *Nr4a1* and *Lrrc17* (Figures 3E and 3F).

We classified RHOX10-regulated genes as “delayed RHOX10-direct targets” if they (1) were bound by RHOX10 (as determined by CUT&Tag) and (2) required at least 2 h exposure to RHOX10-ERT2 and 4OH-TAM to exhibit altered transcription rates (Figures 3E-3G). A plethora of these delayed-target genes encode TFs (*Sox9*, *Bcl6b*, *Ddit3*, *Klf4*, *Lhx2*, *Nr4a1*, *Nr4a2*, *Stat1*, *Stat3*, *Twist2*, and *Bach2*) and thus could participate in networks that regulates batteries of downstream genes. Other classes of delayed-target genes included those encoding factors critical for events in ProSG, including signaling factors (*Klf9*, *Nfkbiz*, *Mcl1*, *Sox9*, *Txnip*, *Hmox1*, *Stat1*, *Stat3*, *Klf4*, *Ddit3*, *Tgif1*, and *Ptpn1*), adhesion proteins (*Ank3*, *Farp2*, *Jak1*, *Lpp*, and *Alcam*), and pro-migration proteins (*Nr4a2*, *Nr4a1*, *Twist2*, *Hmox1*, and *Zswim6*). Many of these genes exhibit sustained altered transcription for many hours after RHOX10 induction; e.g., *Serpine1*, *Mat2a*, *Trib1*, *Nfkbiz*, *Chd3*, *Btg2*, *Sox9*, and *Klf9*.

We classified RHOX10-regulated genes as “secondary RHOX10-response genes” if they (1) were not measurably bound by RHOX10 (as determined by CUT&Tag) and (2) required at least 2 h exposure to RHOX10-ERT2 and 4OH-TAM to exhibit altered transcription rates (Table S2). Many of these secondary-response genes encode TFs (*Dot1l*, *Egr1*, *Egr2*, *Egr3*, *Jun*, *Junb*, *Lhx1*, *Nab2*, *Ssbp3*, *Tead1*, *Cebpd*, and *Sall3*) and RNA-binding proteins (*Cavin1*, *Chtop*, *Fus*, *Junb*, *Ncl*, and *Pim1*) that could serve as regulatory factors influencing ProSG differentiation. Other secondary RHOX10-response genes that could serve in this capacity include those encoding signaling factors (*Abca1*, *Cdkn1a*, *Ddit4*, *Egr1*, *Inhba*, *Klf6*, *Mt1*, *Mt2*, *Nfkbia*, and *Rcan1*), adhesion proteins (*Ext1*, *Tnc*, and *Ccl2*), and pro-migration proteins (*Actg1*, *F2r*, *Egr1*, *Egr3*, *Glul*, and *Lmna*). Secondary-response genes are commonly thought to be regulated by intermediary transcriptional regulators that are directly regulated by the TF of interest (Tullai et al., 2007). In support, we obtained evidence that at least one of the secondary RHOX10-response genes, *Zbtb16*, is regulated by this mechanism (see below).

### RHOX10 regulates the transcription of key SSC genes

**Figure 4.**
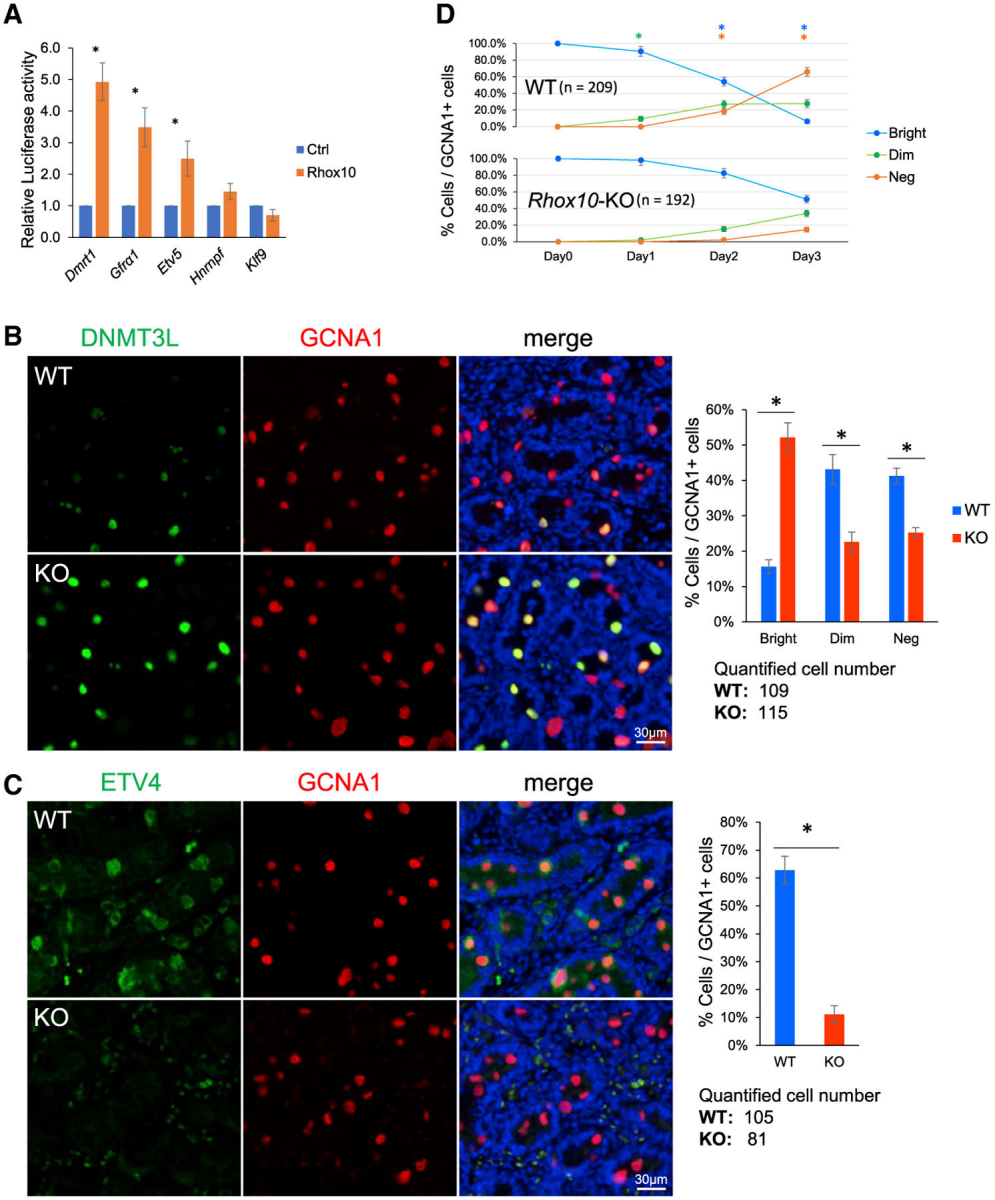
RHOX10 drives ProSG differentiation *in vitro* and *in vivo* (A) Luciferase analysis of constructs harboring promoters from the indicated genes ligated upstream of the Firefly luciferase gene. All promoter constructs contain between 1 to 2 kb of sequence upstream of the TSS (see Table S3). These reporter constructs were transiently co-transfected with an *Rhox10*-expression vector into GC1 cells, which only express trace levels of endogenous *Rhox10*. Data are represented as mean ± SD (n = 4). Staistical significance was determined using the two-tailed unpaired Student’s t test. *p < 0.05. (B) Left, IF analysis of testis sections from P3 *Rhox10*-null (KO) or control (WT) mice co-stained with antisera against DNMT3L and GCNA1, which mark T1-ProSG and all germ cells, respectively. Cell nuclei were stained with DAPI (blue). Scale bar, 30 μm. Right, quantification of the percentage of all germ cells (GCNA1^+^ cells) that express either high (Bright), low (Dim), or no detectable (Neg) DNMT3L. The analysis was done blind to genotype. Statistical significance was determined using the chi-square test. Data are represented as mean ± SD (n = 3). *p < 0.05. (C) Left, IF analysis of testis sections from P3 *Rhox10*-null (KO) or control (WT) mice co-stained with antisera against ETV4 and GCNA1, which stain emergent SSCs and all germ cells, respectively. Cell nuclei were stained with DAPI (blue). Scale bar, 30 μm. Right, quantification of ETV4-positive germ cells, determined as in (B). Data representation and statistical significance (n = 3) as in (B). (D) Quantification of germ cells with the indicated levels of DNMT3L-signal intensity in primary *Rhox10*-null and control testicular cell cultures, analyzed as in (B), at the indicated time points after initiation of culture. Data representation and statistical significance (n = 3) as in (B). See also Figure S4.

Our RNA-seq, CUT&Tag, and iSLAM-seq datasets revealed many interesting genes regulated by RHOX10, as described above. We selected five of these genes—*Gfrα1*, *Dmrt1*, *Etv5*, *Hnrnpf*, and *Klf9*—for further analysis (Figures 2E and 3G). These 5 genes have all been previously shown to function in SSCs or other stem cells, and thus we considered them good candidates to have a role in SSC generation. *Dmrt1* encodes a TF essential for the migration of ProSG to the periphery of the seminiferous tubule (Kim et al., 2007), a developmental process that occurs at approximately the same time as ProSG-to-SSC conversion and brings ProSG into the SSC “niche” (Culty, 2013; McCarrey, 2013). *Etv5* encodes a TF with roles in SSC self-renewal and maintenance; its loss causes progressive germ-cell depletion and Sertoli-cell-only syndrome in mice (Chen et al., 2005). *Gfrα1* encodes a receptor for glial cell-derived neurotrophic factor (GDNF), which is indispensable for SSC self-renewal and maintenance (Meng et al., 2000; Naughton et al., 2006). *Hnrnpf* encodes a RNA-binding protein essential for ESC pluripotency (Yamazaki et al., 2018). *Klf9* is an oxidative stress-inducible gene that encodes a TF expressed in stem cells that regulates the differentiation of many cell types (Ying et al., 2011). The TSS-proximal promoter region of all 5 of these genes is occupied by RHOX10 in germ cells (Figures 2E and 3G). To determine whether RHOX10 is sufficient to regulate the transcription of these genes through their promoters, we cloned their promoters into the pGL3 Luciferase reporter vector. Transfection analysis in the GC1 germ cell line (which largely lacks endogenous RHOX10 [Figure S4A]) showed that forced expression of RHOX10 significantly increased luciferase activity driven by the *Dmrt1*, *Etv5*, and *Gfrα1* promoters (Figure 4A). This is consistent with the downregulation of endogenous *Dmrt1*, *Etv5*, and *Gfrα1* in *Rhox10*-null germ cells *in vivo* (Figure 1C). The *Hnrnpf* promoter also showed a trend toward driving increased luciferase expression in response to RHOX10 (Figure 4A). In contrast, the *Klf9* promoter showed a trend toward being downregulated by RHOX10 (Figure 4A), consistent with the downregulation of endogenous *Klf9* in response to RHOX10 in GS cells (Figure 3G). However, neither *Hnrnpf* nor *Klf9* promoter regulation was statistically significant. To examine generality, we forced expressed RHOX10 in mouse pluripotent P19 cells and found this significantly increased luciferase activity driven by the *Dmrt1*, *Etv5*, *Gfrα1*, and *Hnrnpf* promoters (Figure S4B). We conclude that the promoter regions of *Dmrt1*, *Etv5*, *Gfrα1*, and *Hnrnpf* are sufficient to confer a transcriptional induction response to RHOX10. Since only *Dmrt1*, *Etv5*, and *Gfrα1* were significantly induced by RHOX10 in germ cells, we chose to study these 3 genes in more detail.

### RHOX10 drives ProSG differentiation *in vitro* and *in vivo*

To probe whether the RHOX10-direct target genes, *Dmrt1*, *Etv5*, and *Gfrα1*, have a physiological role downstream of RHOX10, it was critical to examine these genes in the context of ProSG differentiation. ProSG differentiation has traditionally been challenging to study, as, until recently, only one ProSG marker, nuclear FOXO1, was known (Goertz et al., 2011), and there were no known stage-specific ProSG markers (Culty, 2013; McCarrey, 2013). Through scRNA-seq analysis, we recently identified a ProSG-specific protein marker, DNMT3L, whose expression progressively declines as ProSG differentiate, such that few DNMT3L^Bright^ cells remain by the ProSG-to-SSC transition period (Tan et al., 2020c). We confirmed this using immunofluorescence (IF) analysis: testes from wild-type (WT) P3 mice have only ~16% DNMT3L^Bright^ germ cells remaining; most of the germ cells have become DNMT3L^−^ (41%) and the rest are in transition (33% DNMT3L^Dim^) (Figure 4B). In contrast, *Rhox10*-null P3 testes contain 52% DNMT3L^Bright^, 23% DNMT3L^Dim^, and 25% DNMT3L^−^ germ cells (Figure 4B), indicating an accumulation of immature germ cells. This confirms our previous finding—using scRNA-seq analysis—that *Rhox10*-null mice have a defect in ProSG differentiation (Song et al., 2016). To further test this, we performed IF analysis with ETV4, which we recently showed initiates its expression in late T1-ProSG and maintains expression in T2-ProSG and emergent SSCs (Tan et al., 2020c). Consistent with loss of *Rhox10* reducing SSC formation by ~7-fold (Song et al., 2016), *Rhox10*-null P3 testes have ~6-fold lower numbers of ETV4^+^ germ cells than control P3 testes (Figure 4C). Together, these data verify that *Rhox10*-null mice have a defect in ProSG differentiation and SSC formation, thereby demonstrating the utility of the mouse ProSG and SSC markers we identified (Tan et al., 2020c).

In order to define genes that act downstream of RHOX10 to drive ProSG differentiation, it was essential to have an *in vitro* model system. Toward this end, we elected to use P0 testes as a source of germ cells, as this time point is composed of only ProSG (Culty, 2013; McCarrey, 2013), the stage of germ cell that fails to efficiently progress in *Rhox10*-null mice (Song et al., 2016). We isolated testicular cells from P0 testes from both *Rhox10*-null and littermate control mice and cultured them in a previously developed culture medium (Kanatsu-Shinohara et al., 2005). However, the growth factor, GDNF, was omitted to avoid possible confounding effects arising from the fact that the gene encoding its receptor, *Gfrα1*, is one of the genes we planned to functionally test by virtue of it being upregulated by RHOX10 (Figures 1C, 2E, and 4A). For these short-term primary culture experiments, we elected to culture all testicular cells, not just germ cells, as somatic cells in the testes are known to exert essential roles in germ cell development (Oatley and Brinster, 2012; Tan and Wilkinson, 2020).

At the initiation of culture, ~100% of the germ cells were DNMT3L^Bright^ (Figures 4D and S4C; germ cells were marked with GCNA1 [also known as TRA98]), as expected, given that all germ cells from P0 mice are known to be ProSG (Culty, 2013; McCarrey, 2013). Consistent with the timing of the T1- to T2-ProSG conversion event *in vivo*, which occurs at ~P2 (Culty, 2013; McCarrey, 2013), the proportion of DNMT3L^Bright^ germ cells in these P0 testicular cell cultures declined to 54% at day 2 and only 6% at day 3 (Figures 4D and S4C). This *in vitro* temporal expression pattern of DNMT3L mimics that which occurs *in vivo* (Figure 4B). In marked contrast, the *Rhox10*-null cultures failed to show this dramatic reduction of DNMT3L^Bright^ cells, with almost no reduction at day 2 (Figures 4D and S4C). Even by day 3, most of the *Rhox10*-null germ cells remained DNMT3L^Bright^ (51%); some were DNMT3L^Dim^ (34%) and only 15% had become DNMT3L^−^ cells (Figures 4D and S4C). These *in vitro* results recapitulate the effect of *Rhox10* knockout *in vivo* (Figure 4B), thereby validating our *in vitro* system.

### RHOX10 acts through DMRT1 to drive ProSG differentiation

**Figure 5.**
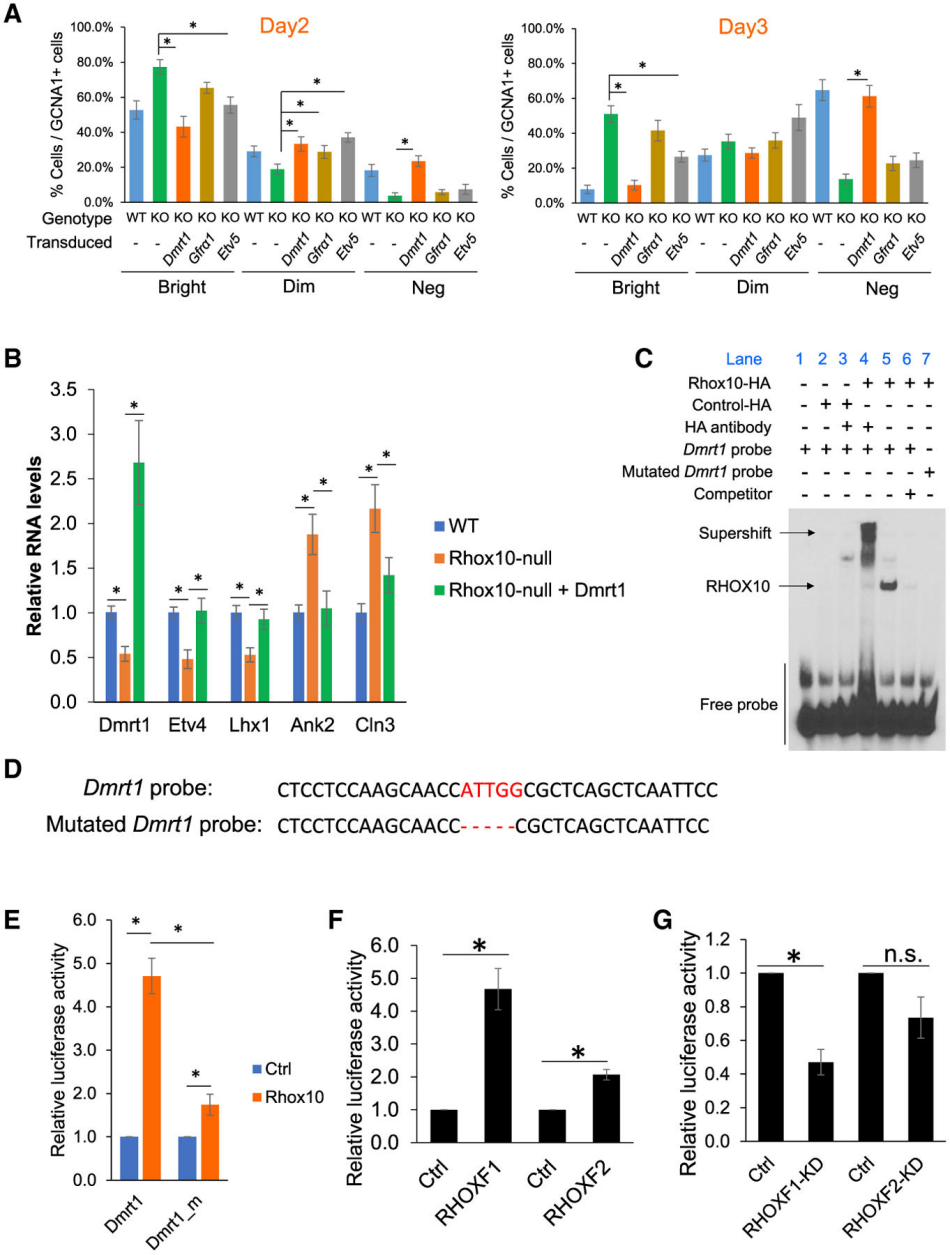
RHOX10 drives ProSG differentiation through transcriptional activation of *Dmrt1* (A) Quantification of the percentage of all germ cells (GCNA1^+^ cells) in *Rhox10*-null (KO) and control (WT) primary testicular cell cultures that express either high (Bright), low (Dim) or no detectable (Neg) DNMT3L, performed as in Figures 4B-4D. The analysis was done blind to genotype. Data are represented as mean ± SD (n = 3). Statistical significance was determined using the chi-square test. *p < 0.05. *Dmrt1*, *Gfrα1*, and *Etv5* were transduced ectopically using lentiviral expression vectors. (B) qPCR analysis of testicular primary cultures of the indicated genotype transduced with or without a *Dmrt1*-expression vector. Data representation as in (A). Statistical significance was determined using the Student’s t test (n = 3). (C) EMSA showing that RHOX10 binds to the predicted RHOX10-binding site ATTGG (reverse complement of CCAAT in Figure 2C) in the *Dmrt1* promoter region. The competitor is unlabeled *Dmrt1* promoter probe (n = 3). (D) The *Dmrt1* and mutated *Dmrt1* probes used for EMSA in (C). (E) Luciferase analysis of constructs harboring either the *Dmrt1* promoter or a mutated version of the *Dmrt1* promoter lacking the CCAAT motif (*Dmrt1_m*) ligated upstream of the Firefly luciferase gene. These constructs were transiently co-transfected into GC1 cells with a *Rhox10*-expression vector where indicated. Data representation and statistical significance (n = 3) as in (B). (F) Luciferase analysis of a construct harboring the human *DMRT1* promoter ligated upstream of the Firefly luciferase gene. This construct was transiently co-transfected into TCam-2 cells with the *RHOXF1*- or *RHOXF2*-expression vectors, as indicated. Data representation and statistical significance (n = 3) as in (B). (G) Luciferase analysis of TCam-2 cells transiently co-transfected with the human *DMRT1* promoter construct (described in F) with either a *RHOXF1*- or *RHOXF2*-shRNA vector (RHOXF1-KD and RHOXF2-KD, respectively). Data representation and statistical significance (n = 3) as in (B).

With the *in vitro* culture system we developed above, coupled with the ProSG and SSC markers we identified, we were in a position to ask whether *Dmrt1*, *Gfrα1*, or *Etv5* act downstream of RHOX10 to drive ProSG differentiation. To address this question, we first performed a rescue experiment in which we force expressed *Dmrt1*, *Gfrα1*, or *Etv5* in *Rhox10*-null primary cells. The rationale was that if any of these 3 genes requires being transcriptionally activated by RHOX10 to drive ProSG differentiation, then rescue of their expression in *Rhox10*-null cells should also rescue ProSG differentiation. Figure 5A shows that forced expression of *Dmrt1* did indeed rescue ProSG differentiation, leading to almost complete loss of DNMT3L^Bright^ cells (ProSG) in *Rhox10*-null testicular cultures by day 3, analogous to WT cells (Figure 5A). Forced expression of the other two genes we tested, *Gfrα1* and *Etv5*, also led to some rescue of ProSG differentiation, but this was only evident in certain cell populations and time points, e.g., DNMT3L^Dim^ at day 2 (Figure 5A). In contrast, *Dmrt1* had statistically significant robust effects in most subsets at both time points (Figure 5A), and thus we elected to only pursue investigating *Dmrt1* as a downstream mediator of RHOX10.

To further test whether *Dmrt1* rescues the ProSG differentiation defect caused by loss of RHOX10, we performed qPCR analysis on germ cells purified from the testicular cultures using EpCAM, an early germ cell marker (Inoue et al., 2017; Watanabe et al., 2018). We tested two classes of markers: those that go up during the ProSG-to-SSC transition *in vivo* (*Etv4* and *Lhx1*) and those that go down during this transition (*Ank2* and *Cln3*) (Tan et al., 2020c). These 4 markers responded as expected in *Rhox10*-null germ cells: *Etv4* and *Lhx1* were reduced relative to control germ cells, indicative of perturbed progression to SSCs, while *Ank2* and *Cln3* were elevated, indicative of accumulation of ProSG (Figure 5B). Forced expression of *Dmrt1* in *Rhox10*-null testicular cells rescued the expression of all 4 of these markers, such that their levels were not statistically different from WT (Figure 5B), thereby verifying that *Rhox10* drives ProSG differentiation through transcriptional activation of *Dmrt1*.

While the above collective results suggest that RHOX10 drives the ProSG-to-SSC transition by directly transcriptionally activating *Dmrt1* (Figures 1C, 2E, and 4A), we sought to solidify this finding by testing whether RHOX10 binds to *Dmrt1* promoter DNA sequences (the CUT&Tag technique we used in Figure 2 measures TF occupancy in chromatin, not direct DNA binding). To this end, we elected to use the electrophoretic mobility shift assay (EMSA) (Garner and Revzin, 1981). EMSA showed that an oligonucleotide corresponding to the region of *Dmrt1* with high RHOX10 occupancy as defined by CUT&Tag (Figure 2E) exhibited retarded mobility in response to HA-tagged RHOX10 (Figure 5C, lane 5). As confirmation, cold competitor oligonucleotide greatly reduced the signal (Figure 5C, lane 6) and addition of anti-HA antibody elicited a supershift (Figure 5C, lane 4). The predicted RHOX10-binding sequence in this *Dmrt1* promoter region is CCAAT (reverse complement of #2 predicted RHOX10-binding motif in Figure 2C). To test its role, we deleted this sequence from the oligonucleotide and found this prevented RHOX10 binding (Figure 5C, lane 7; Figure 5D).

To test whether RHOX10 requires this CCATT motif to elicit transcription induction of *Dmrt1*, we removed this motif from the *Dmrt1* promoter-luciferase reporter we used for Figure 4A. Transfection analysis showed that this mutated reporter had a greatly blunted response to RHOX10 (Figure 5E). Together, these results demonstrated that RHOX10 directly binds to a CCAAT motif in the *Dmrt1* promoter region and that this binding confers *Dmrt1* transcriptional induction in response to RHOX10.

*Dmrt1* is a highly conserved gene in vertebrates (Raymond et al., 2000). Thus, we elected to examine whether the ability of RHOX TFs to regulate *Dmrt1* extends to humans. The human genome contains three *RHOX* genes—*RHOXF1*, *RHOXF2*, and *RHOXF2B* (Niu et al., 2011)—the latter two are likely to be functionally equivalent, as they are 99.8% identical in sequence in their exons and introns (Wayne et al., 2002). Both RHOXF1 and RHOXF2 are exclusively expressed in germ cells in humans (Song et al., 2013). In particular, both are expressed in ProSG in human fetal testes (Song et al., 2013) and thus have the potential to regulate human *DMRT1* in the context of ProSG differentiation. To address whether human RHOX TFs regulate human *DMRT1*, we cloned the human *DMRT1* promoter region into the pGL3 Luciferase reporter vector and transfected this into the human TCam-2 germ cell line. This revealed that forced expression of either *RHOXF1* and *RHOXF2* significantly increased luciferase activity driven by the *DMRT1* promoter (Figure 5F). *RHOXF1* exerted a stronger effect than *RHOXF2*. Conversely, RNAi-mediated *RHOXF1* knockdown significantly decreased luciferase activity driven by the *DMRT1* promoter (Figure 5F). *RHOXF2* knockdown elicited a trend toward decreased luciferase activity but it was not statistically significant (Figure 5G). To examine cell-type specificity, we forced expressed *RHOXF1* and *RHOXF2* in HEK293T cells and found that the former, but not the latter, significantly increased luciferase activity driven by the *DMRT1* promoter (Figure S4D). We conclude that the ability of RHOX TFs to transcriptionally activate *Dmrt1/DMRT1* is a conserved response.

### A RHOX10-DMRT1-ZBTB16 regulatory circuit drives ProSG differentiation and SSC generation

**Figure 6.**
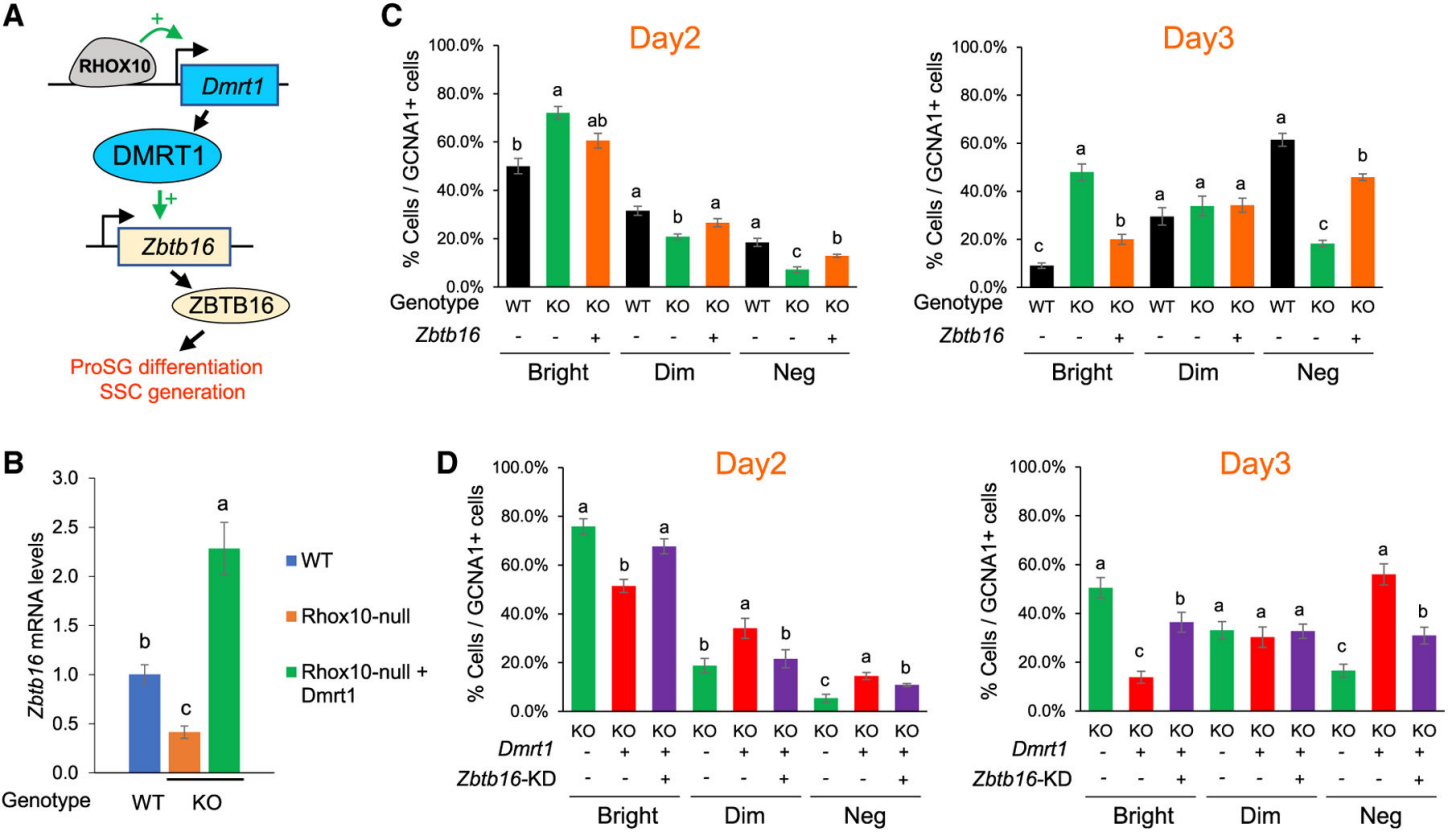
A RHOX10-DMRT1-ZBTB16 circuit driving ProSG differentiation (A) Model: the RHOX10 TF drives ProSG differentiation and consequent SSC production through a TF cascade involving DMRT1 and ZBTB16. (B) qPCR analysis of *Zbtb16* expression in germ cells purified from primary testicular cell cultures (described in Figures 4D and 5A) of the indicated genotype, and transduced with a *Dmrt1*-expression vector, where indicated. Data are represented as mean ± SD (n = 3). Statistical significance was determined using the Student’s t test. Different letters (a, b, c) denote statistically significant differences between different groups (p < 0.05). (C and D) Quantification of the percentage of all germ cells (GCNA1^+^ cells) in *Rhox10*-null (KO) and control (WT) primary testicular cell cultures that express either high (Bright), low (Dim), or no detectable (Neg) DNMT3L, performed as in Figures 4 and 5. The analysis was done blind to genotype. *Dmrt1*- and *Zbtb16*-expression vectors were transduced where indicated. *Zbtb16* was knocked down by transduction of a *Zbtb16* shRNA construct (*Zbtb16*-KD). Statistical significance was determined using the chi-square test. Data are represented as mean ± SD (n = 3). Different letters (a, b, c) denote statistically significant differences between different groups (p < 0.05).

The experiments delineated above provided many lines of evidence that a RHOX10-DMRT1 TF regulatory circuit promotes ProSG differentiation. We considered the possibility that other TFs may also participate in this circuit. One promising candidate was ZBTB16, a zinc-finger TF also known as PLZF that has been shown to be positively regulated by DMRT1 in mouse male germ cells *in vivo* (Zhang et al., 2016). Given this regulatory relationship between DMRT1 and ZBTB16, we hypothesized that RHOX10 drives ProSG differentiation and SSC formation via a regulatory circuit in which RHOX10 activates *Dmrt1* transcription, generating DMRT1 protein, which, in turn, activates *Zbtb16* transcription, thereby generating ZBTB16 protein, which drives ProSG differentiation (Figure 6A). Several lines of evidence supported this hypothesis: first, inspection of our RNA-seq data revealed that *Zbtb16* is positively regulated by RHOX10 (Table S1), which we validated by qPCR (Figure 6B). Second, Zbtb16 does not have a detectable RHOX10 occupancy site (Table S1), suggesting it is indirectly regulated by RHOX10. Third, forced expression of *Dmrt1* in *Rhox10*-null germ cells upregulated *Zbtb16* expression (Figure 6B). Fourth, forced expression of *Zbtb16* in *Rhox10*-null testicular cell cultures rescued the ability of these knockout cells to undergo ProSG differentiation (Figure 6C). Finally, *Zbtb16* knockdown in *Dmrt1*-overexpressing *Rhox10*-null testicular cell cultures prevented ProSG differentiation (Figure 6D). Together, these experiments provide several lines of molecular and functional lines of evidence for a RHOX10-DMRT1-ZBTB16 TF regulatory circuit that drives SSC establishment (Figure 6A).

## DISCUSSION

Homeobox genes were originally discovered in flies as key developmental regulators whose loss causes conversion of one body part into another (homeosis), as well as other striking developmental abnormalities (Garber et al., 1983). All homeobox proteins harbor a triple α-helical 60 amino-acid DNA-binding domain referred to as a homeodomain. The *Hox* subfamily of homeobox genes was the first to be discovered and have since been shown to encode TFs that control several discrete steps in embryonic development across the phylogenetic scale (Heffer et al., 2013). However, many other homeobox gene subfamilies exist; indeed, mammalian species have over 200 homeobox genes, many of which are not well understood, including the subject of this report: the members of the X-linked *Rhox* cluster. Indeed, the biological roles of *Rhox* genes are largely unknown. While some of the 33 mouse *Rhox* genes have been knocked out or depleted, most of the resulting phenotypes are subtle or undetectable (Brown et al., 2013; Busada et al., 2016; Hu et al., 2010; Maclean et al., 2005, 2013; Takasaki et al., 2001). Furthermore, to date, only one RHOX protein—RHOX5—has been demonstrated to be a TF; albeit only one direct target was defined (MacLean et al., 2013). In this communication, we focus on *Rhox10*, the only Rhox gene whose loss causes major spermatogenic defects, including inefficient SSC generation and progressive loss of spermatogenesis (Song et al., 2016). We show here that *Rhox10* encodes a TF that regulates an intriguing array of targets, including many genes previously known to function in SSCs. Using a battery of genome-wide approaches, we identify both direct and likely secondary targets of RHOX10 in germ cells. We also modified a genome-wide metabolic labeling approach to permit temporal analysis of TF targets, which allowed us to define waves of RHOX10 target genes, including both immediate early and delayed targets encoding scores of other TFs. Finally, using a primary culture system we developed, coupled with stage-specific germ-cell markers we identified, we obtained evidence that RHOX10 participates in a TF cascade driving the ProSG-to-SSC transition.

Our discovery that a RHOX10-DMRT1-ZBTB16 TF cascade participates in SSC establishment not only advances our understanding of RHOX10 but also DMRT1 and ZBTB16, both of which have been examined in detail in past studies. Most of what is known about DMRT1 is its roles in driving the differentiation, survival, and proliferation of germ cells at later stages than those studied herein (at P7 and later postnatal time points in mice) (Fahrioglu et al., 2007; Kim et al., 2007; Raymond et al., 2000). DMRT1 has also been shown to be involved in the maintenance of SSCs in adult testes (Zhang et al., 2016). With regard to ProSG, DMRT1 has been shown to be required for the efficient migration of these cells from the center of the seminiferous tubule to the stem cell niche at the periphery (Kim et al., 2007). This ProSG migration defect phenocopies that observed in *Rhox10*-null mice (Song et al., 2016), supporting our finding that RHOX10 positively regulates *Dmrt1*. ZBTB16 was previously shown to have roles in SSCs, based on analysis of *Zbtb16* mutant mice (Buaas et al., 2004; Costoya et al., 2004). Consistent with *Zbtb16* acting downstream of *Rhox10*, the testicular phenotype of *Zbtb16* mutant mice is similar in many respects with that of *Rhox10*-null mice, including progressive spermatogenic failure, resulting in regions of the seminiferous tubule devoid of early germ cells or all germ cells (Buaas et al., 2004; Costoya et al., 2004). While this was ascribed to be the result of a failure in SSC maintenance, a notion supported by more recent work (Sharma et al., 2019), the spermatogenic abnormalities in *Zbtb16* mutant mice may also result from a failure to generate a normal pool of SSCs. Consistent with this, *Zbtb16*-mutant mice exhibit inefficient expansion of the germ cell pool during the postnatal period (Costoya et al., 2004). Strikingly, *Zbtb16* mutant mice have an ~7-fold reduction in Oct4-GFP^+^ (SSC-enriched) cells at P8 (Buaas et al., 2004), analogous with the ~6-fold reduction in SSCs in P7–P8 *Rhox10*-null mice, as measured by germ cell transplantation (Song et al., 2016). This raises the possibility that, like *Rhox10*-null mice, *Zbtb16* mutant mice have a defect in SSC establishment, a notion supported by the fact that ZBTB16 protein is readily detectable at the ProSG stage (Costoya et al., 2004) and our finding that ZBTB16 acts downstream of RHOX10 and DMRT1 to drive the differentiation of ProSG *in vitro*. The hierarchical regulatory relationship of the RHOX10, DMRT1, and ZBTB16 TF triade, with RHOX10 most upstream, suggests that RHOX10 has the potential to influence many biological events in developing germ cells.

We designed an approach—iSLAMseq—to identify RHOX10-direct targets and to determine whether there are temporal waves of genes undergoing alterations in transcription rate in response to RHOX10. We suggest this iSLAMseq approach will have broad applications for studying other TFs. As a testament to its usefulness, we found that 31%–44% of iSLAMseq-defined RHOX10-regulated gene had RHOX10 bound (as determined by CUT&Tag), which was higher than the proportion of RNA-seq-defined DEGs with RHOX10 occupancy (23%). Among the RHOX10-regulated genes defined by iSLAMseq are many TF genes (see Results), raising the possibility that RHOX10’s reach extends well beyond the *Dmrt1* and *Zbtb16* TF genes.

iSLAMseq coupled with CUT&Tag allowed us to define direct target genes undergoing very rapid shifts in transcription rate in response to RHOX10. Among these RHOX10-direct target genes was Paip2, which encodes a translational repressor protein (Khaleghpour et al., 2001), whose loss leads to male infertility (Yanagiya et al., 2010). Other immediate-early targets encode proteins with interesting functions in non-germ cell types, including the TF NR4A1 and the thioredoxin-binding protein TXNIP, both of which act in the hematopoietic system, including hematopoietic stem cells (Freire and Conneely, 2018; Jung et al., 2013), and the RNA-binding protein HNRNPF, which modulates telomerase function (Xu et al., 2020). iSLAMseq also allowed us to identify genes that respond to RHOX10 with delayed kinetics. We defined those with RHOX10 occupancy as delayed-direct targets, while those not significantly occupied by RHOX10 as secondary-response targets. An intriguing possibility is that a subset of these secondary-response genes could participate in TF circuits analogous to the RHOX10-DMRT1-ZBTB16 TF cascade we showed has a functional role in germ-cell differentiation. In support, we found that many RHOX10-direct targets encode TFs and thus are logical candidates to be responsible for regulating a subset of the RHOX10 secondary-response genes. The identification of genes with different temporal kinetics in response to RHOX10 *in vitro* may also reflect what occurs in developing germ cells *in vivo*. For example, delayed RHOX10-mediated regulation would allow turn-on and -off of specific functions requiring a temporal delay after RHOX10 induction *in vivo*.

The number of RHOX10 direct targets we identified using iSLAMseq may be an underestimate. Part of this may be due to limited sensitivity given the amount of sample we used. Another contributing factor may be that we only classified RHOX10-regulated genes as direct targets if they were also bound by RHOX10, as detected by CUT&Tag. Some of the genes that scored as “negative” may be sufficiently bound by RHOX10 to trigger a transcriptional response. We suspect this is the case, as more than 1/2 of the immediate-early RHOX10 direct-target genes (those transcriptionally regulated very rapidly after 4OH-TAM treatment (after 0.5 h or 1 h) to induce RHOX nuclear translocation) were not scored as having RHOX10 occupancy. We suspect that many of these genes are direct targets, which would expand the list of direct target genes, including genes with well-established functions, e.g., *Ubc*, *Egr1*, *Eef1a1*, *Brd2*, *Ptgs2*, *Cdkn1a*, *Phlda1*, *Actg1*, *Ssbp3*, *Cxcl1*, *Actb*, and *Rpl41* (Table S2). For the same reason, we cannot distinguish—with certainty—which RHOX10-regulated genes are delayed direct targets versus secondary-response targets. Those with RHOX10 occupancy are likely direct targets and those not detectably bound by RHOX10 will often be secondary response genes, but proof would require detailed further investigation on a gene-by-gene basis. Nonetheless, our analysis identified many high-confidence TF-direct targets—in a rare cell population *in vivo*—that displayed either immediate or delayed responsiveness. All of these high-confidence RHOX10 target genes are detectably expressed in purified P1 germ cells (Table S1).

An important future question is to determine whether RHOX TFs have a conserved role in human SSC establishment. In support of this possibility, both human RHOXF1 and RHOXF2 protein are expressed in germ cells in human fetal and adult testes (Song et al., 2013). Furthermore, we showed here that both human RHOXF1 and RHOXF2 can induce *DMRT1* in human germ cells. However, to definitively address this question, it is necessary to first determine when SSCs first form in humans. While human SSC-like cells have been detected by scRNA-seq analysis at birth (Sohni et al., 2019) and postnatally (Guo et al., 2020), precisely when functional self-renewing human SSCs are first generated is not known. Understanding how human SSCs first form may provide important insights into how to culture and expand human SSC for clinical applications in treating infertility (Tan and Wilkinson, 2020). In this light, it will be intriguing to determine whether genes induced by the RHOX10-DMRT1-ZBTB16 TF cascade participate in signaling pathways that permit the culture and expansion of SSCs *in vitro*.

## STAR★METHODS

### RESOURCE AVAILABILITY

#### Lead contact

Further information and requests for resources and reagents should be directed to and will be fulfilled by the Lead Contact, Miles F. Wilkinson (mfwilkinson@health.ucsd.edu).

#### Materials availability

This study did not generate new unique reagents. All the materials used in this study are preserved by Dr. Miles F. Wilkinson’s laboratory and are available upon request. *Rhox10* null mouse line (B6.129-*Rhox10*^tm1Wilk^/J) used in this study has been deposited to the Jackson Laboratory (JAX: 035193).

#### Data and code availability

The RNaseq, CUT&Tag, and iSLAMseq data generated in this study have been deposited at the NCBI GEO database and are publicly available as of the date of this publication. Accession numbers are listed in the key resources table. Microscopy data reported in this paper will be shared by the lead contact upon request.

No unpublished custom code, software or algorithm was used in this study. Freely available software and algorithms used for analysis are listed in the Key Resources Table. All original code will be shared by the lead contact upon request.

Any additional information required to reanalyze the data reported in this paper is available from the lead contact upon request.

### EXPERIMENTAL MODEL AND SUBJECT DETAILS

#### Mice

This study was carried out in strict accordance with the guidelines of the Institutional Animal Care and Use Committee (IACUC) at the University of California, San Diego. The protocol was approved by the IACUC at the University of California, San Diego (permit number: S09160). All mice were housed under a 12 hr light:12 hr dark cycle and provided with food and water *ad libitum*. All mouse strains used for analysis were backcrossed to C57BL/6J for at least 8 passages. As described previously (Song et al., 2016), floxed-*Rhox10* mice were mated with *Ella-Cre* mice to generate global *Rhox10*-KO mice. For mating, males (aged 8-16 weeks) and females (8-16 weeks) were used. Testes from P0-P2 male pups were collected for experiments, as described below.

#### Primary cell cultures and cell lines

GC1 and HEK293T cells were grown in DMEM (GIBCO), 10% fetal bovine serum (sigma), and 1× penicillin/streptomycin (GIBCO). P19 cells were grown in MEMα (GIBCO), 10% fetal calf serum, and 1× penicillin/streptomycin (GIBCO). TCam-2 cells (de Jong et al., 2008) were grown in advanced RPMI 1640 medium (GIBCO), 10% fetal calf serum, 2 mM L-glutamine, and 1× penicillin/streptomycin (GIBCO). Mouse GS cells were prepared by dissociating single cell from neonatal (P0-P2) testes as previously described (Kanatsu-Shinohara et al., 2003). In brief, testicular cells were collected by two-step enzymatic digestion (see details in the Testicular Cell Dissociation section, below), plated on 0.1% gelatin-coated plates, and incubated overnight. Germ cells were enriched by gelatin selection by collecting non-attached cells by gentle pipetting and transferring them to laminin (Corning)-coated plates (8 μg/3.8 cm^2^), and cultured in IMDM/SFM, as described previously (Kanatsu-Shinohara et al., 2014). The IMDM/SFM media composition is: Iscove modified Eagle medium (IMDM) supplemented with 25 μg/ml insulin (Sigma), 100 μg/ml apo-Transferrin (sigma), 200 μg/ml sodium pyruvate (Thermo Fisher Scientific), 60 μM putrescine (Sigma), 30 nM sodium selenite (Sigma), 6 mg/ml D-(+)-glucose (Sigma), 1 μl/ml DL-lactic acid (sigma), 5 mg/ml bovine albumin (Sigma), 2 mM GlutaMAX supplement (Thermo Fisher Scientific), 50 μM 2-mercaptoethanol (Thermo Fisher Scientific), 1× MEM vitamin solution (Thermo Fisher Scientific), 1× non-essential amino acids (Thermo Fisher Scientific), 100 μM ascorbic acid (Sigma), 10 μg/ml d-Biotin (Sigma), 30 ng/ml β-Estradiol (sigma), 60 ng/ml progesterone (Sigma), 1 mg/ml fetuin (Sigma), 10 μl/ml CD Lipid concentrate (Thermo Fisher Scientific), 2 μl/ml cholesterol solution (Sigma), and 50 μl/ml knockout serum replacement (Thermo Fisher Scientific), 10 ng/ml bFGF (Sigma), and 15 ng/ml recombinant human GDNF protein (R&D Systems). All cells were cultured in a humidified 5% CO_2_, 37°C incubator.

### METHOD DETAILS

#### Transfection and viral transduction

For reporter assay, the promoter regions of candidates were cloned into pGL3 vector (Promega). The primers for generating these reporters are listed in Table S3. The *RHOXF1*- and *RHOXF2*-shRNA plasmids were generated by inserting ologonucleotides corresponding to siRNA sequences (Table S3) into the pLLU2G backbone. HEK293T cells were transfected with the Lipofectamine 2000 reagent (Invitrogen). GC1 and TCam-2 cells were transfected with the Lipofectamine 3000 reagent (Invitrogen). GS cells were transfected with the Lipofectamine stem reagent (Invitrogen). For transfection experiments, most cells were trypsinized and seeded in 24-well plates at a density of ~70,000 cells per well. TCam-2 cells were seeded at 20,000 cells/well. Transfection was performed following the manufacturer’s instructions. For lentiviral transduction, 1 infectious unit (IU) of lentivirus per cell was added with 6 μg/ml polybrene. Cells were harvested 1 day post-transfection for luciferase activity analysis. Luminescence was measured using the Dual-Luciferase Reporter assay system (Promega) following the manufacturer’s instruction. The pRL-cmv (Renilla) vector was co-transfected in these experiments as an internal control for normalization. Statistical significance was determined using the paired Student’s t-test.

#### qRT-PCR analysis

Total cellular RNA was isolated using TRIzol (Invitrogen), as previously described (Ramaiah et al., 2019). Reverse transcription-PCR analysis was performed using 1 μg of total cellular RNA using iScript cDNA synthesis kit (Bio-Rad), followed by PCR amplification using SYBR Green (Bio-Rad) (Tan et al., 2016a) and the ΔΔCt method (with ribosomal L19 for normalization). The primers are listed in Table S3. Statistical significance was determined using the paired Student’s t-test.

#### Immunofluorescence analysis

As previously described (Tan et al., 2020a; Tan et al., 2020c), testes were fixed in Bouin’s fixative for 0.5 h at room temperature, and then cleared using multiple changes of 70% ethanol and kept at 4°C. Tissues were dehydrated through an ethanol series—30%, 50%, 70%, 95%, 100%, and 100%—for 10 min each at room temperature, and cleared with two changes of xylene and embedded in paraffin. Tissue blocks were sectioned at 5 μm. Sections were deparaffinized twice in xylene, followed by serial dilutions of ethanol. Unmasking was performed using a steamer (IHC World). Blocking was performed by incubating with 5% goat or donkey serum (Sigma), depending on the usage of secondary antibodies, for 1 hr at room temperature. The sections were then incubated overnight with the primary antibody (listed in Table S3) at 4°C and incubated with secondary antisera (listed in Table S3) for 1 hr at room temperature. The nuclei were counterstained with DAPI (Vector Laboratories), a coverslip was placed over the sections with mounting medium, and the images were viewed using a Leica DMI4000 B fluorescence microscope. Statistical significance was determined using the chi-square test.

#### Testicular cell dissociation

Single testicular cells were isolated from P0 or P2 testes using a two-step enzymatic digestion protocol previously described (Tan et al., 2020c), with minor modifications. In brief, testicular tissue was mechanically disrupted and enzymatically digested with 1 mg ml^−1^ collagenase type IV (Worthington Biochemical) in Hanks Balanced Salt Solution (HBSS; GIBCO) at 37°C. The tubules were sedimented and washed with HBSS and digested in 0.25% Trypsin-EDTA (ThermoFisher) and Deoxyribonuclease I (Worthington Biochemical). The suspension was triturated vigorously ten times, incubated at 37°C for 5 min, followed by repeat trituration and incubation. The digestion was stopped by adding the same volume of αMEM + 10% FBS medium and the cells were size-filtered through 70 μm strainers (ThermoFisher), and pelleted by centrifugation at 300 g for 5 min.

For *in vitro* culture, testicular cells were isolated from P0 testes from both *Rhox10*-null and littermate control mice. The total cell suspensions were cultured using a modified version of the early postnatal germ-cell culture medium (IMDM/SFM) developed by Kanatsu-Shinohara et al. (Kanatsu-Shinohara et al., 2005). The main modification we made is to omit GDNF, as described in the Results section.

#### RNA-seq analysis

To obtain the samples for RNA-seq analysis, wt C57BL/6J mice were crossed with *Oct4*-eGFP mice. After isolating single cells as described above, eGFP+ cells were sorted by FACS. For each sample analyzed, testes from 3 individuals were pooled. Four replicate samples were analyzed per genotype. RNA-seq was performed as described previously (Tan et al., 2020b; Tan et al., 2021). Total RNA was extracted using the RNeasy Plus Micro Kit (QIAGEN), following the manufacturer’s protocol. 30 ng of total RNA was used to make the library using the NEBNext Ultra II Directional RNA Library Prep kit (New England Biolabs), as per manufacturer’s instructions. Libraries were sequenced (pair-end reads) with an Illumina HiSeq 4000 platform for 100 cycles at the UCSD institute for Genomic Medicine (IGM) core. Reads were filtered for quality and aligned with STAR (2.5.2b) (Dobin and Gingeras, 2015) against the *Mus musculus*, release-96, Ensembl genome (GRCm38). Exon counts were aggregated for each gene to build a read count table using SubRead function featureCounts (Liao et al., 2013). DEGs were defined using DESeq2 (Love et al., 2014) using the following threshold: ∣Log2FC∣ > 0.5, and *q*-val < 0.01. The R package program “pheatmap” was used for clustering and generating heatmap plots. The database for annotation, visualization and integrated discovery (DAVID) v6.8 was used for signaling pathway analysis (Huang da et al., 2009; Ren et al., 2015; Tan et al., 2016b).

#### CUT&Tag analysis

Endogenous *Rhox10* was knocked-down (~85%) in GS cells using an shRNA lentivirus and the cells purified on the basis of GFP fluorescence by FACS. The *Rhox10* shRNA used is specific to *Rhox10*, as reported previously (Song et al., 2012). The shRNA is complementary with sequences encoding a unique N-terminal region only present in RHOX10 (upstream of the homeodomain common to all RHOX proteins). The cells were then transduced with a mCHERRY reporter lentivirus that also expresses HA-tagged RHOX10 from a form of *Rhox10* harboringnucleotide substitutions that make it insensitive to the *Rhox10* shRNA without changing the protein sequence it encodes. CUT&Tag was performed as previously described (Kaya-Okur et al., 2019). ~1 million GS cells were used per sample and three biological replicates were performed. As a negative control, the lysates were instead incubated with IgG sera (Table S3). Samples from *Rhox10*-kd; HA-tagged empty vector GS cells served as another negative control. In brief, GS cells were bound to Concanavalin A beads (Bangs Laboratories), permeabilized with 0.05% digitonin (EMD Millipore), and incubated with an anti-HA antibody (Table S3) overnight at 4°C on a nutator. After washing, the secondary antibody (Guinea Pig anti-Rabbit IgG; Table S3) was incubated at room temperature for 1 hr, followed by incubation with Protein A-fused Tn5 transposase (pA-Tn5; a gift from Dr. Steven Henikoff laboratory) for 1 hour at 37°C. The DNA from the cells was extracted and purified. Libraries were dual-indexed and amplified with NEBNext HiFi 2× PCR Master mix (New England BioLabs: M0541L). Libraries were sequenced (pair-end reads) with an Illumina HiSeq 2500 platform for 50 cycles at the UCSD IGM core.

For analysis, reads were filtered for quality and aligned with bowtie2 (v2.2.5) against the *Mus musculus* (mm10) genome, with the following parameters: –local–very-sensitive-local–no-unal–no-mixed–no-discordant–phred33 -I 10 -X 700, as described previously (Meers et al., 2019). Following alignment, multiple-aligned reads were discarded and read duplicates were removed by samtools rmdup using default parameters (Li et al., 2009). Normalization to GC-content was performed using Homer (Heinz et al., 2010), and peak calling was performed using Homer *findPeaks* function with “-style factor” mode (Heinz et al., 2010). Motif finding and annotation were performed using Homer *findMotifsGenome.pl* and *annotatePeaks.pl* functions, respectively (Heinz et al., 2010), applying cumulative hypergeometric distribution adjusted for multiple testing with the Benjamini-Hochberg method. Track screen shots were produced in IGV (version 2.6.2) (Robinson et al., 2011).

#### iSLAMseq ANALYSIS

Endogenous *Rhox10* in mouse GS was knocked-down (~85%) using a shRNA lentivirus (Song et al., 2012) and purified on the basis of GFP fluorescence by FACS. Also transduced was a lentivirus expression vector carrying a *Rhox10*-ERT2-mCherry fusion gene that confers conditional inducible *Rhox10* expression in response to 4OH-TAM.

SLAMseq was performed as previously described (Herzog et al., 2017). Briefly, ~2 millions GS cells were incubated with 200 nM 4OH-TAM for different time periods (0 min, 30 mins, 1 hr, 2 hr, 4 hr, and 8 hr), as indicated in Figure 3A. 4OH-TAM was dissolved in ethanol; ethanol only was used for the control group. Before collecting samples, cells were pulse-treated with 500 μM s^4^U for 15 mins. After extracting total RNA, carboxyamidomethylation was performed under standard conditions (~10 μg RNAs, 50% DMSO, 10 mM IAA, 50 mM sodium phosphate buffer pH 8, incubated for 20 min at 50°C). The reaction was quenched by addition of 1 μL of 1M DTT. After purifying the RNA, libraries were prepared using the Quant-seq mRNA 3′ end library preparation kit (Lexogen) according to the manufacturer’s instructions. Sequencing was performed using a Illumina HiSeq 4000 at the UCSD IGM core in the SR75 mode.

Reads were filtered for quality and aligned with SLAM-DUNK (v0.4.3) (Neumann et al., 2019) against the full *Mus musculus* reference genome (mm10), reporting up to 100 alignments for multi-mappers and activating the multi-mapper retention strategy, filtering for variants with a variant fraction of 0.2. Gene and 3′ UTR annotations were obtained from the UCSC table browser (https://genome.ucsc.edu/cgi-bin/hgTables). T > C conversion rate was determined for each position along the custom defined counting windows by normalizing to genomic T content and coverage of each position and averaged per UTR. Differential gene expression calling was performed on raw read counts with ≥2 T > C conversions using limma (v3.42.2) (Ritchie et al., 2015).

#### EMSA

3′-biotinylated oligonucleotides (listed in Table S3) were labeled using the Pierce biotin 3′ end DNA labeling kit (Thermo Fisher Scientific). Nuclear extracts from GC1 cells transduced with *Rhox10*-HA lentivirus were prepared as previously described (Abmayr et al., 2006). Competition and supershift assays were performed using the LightShift Chemiluminescent EMSA kit (Thermo Fisher Scientific), following the manufacturer’s protocol. Briefly, 1X binding buffer, 1 μL of 50% glycerol, 1 μL of 100mM MgCl_2_,1 μg Poly (dl·dC), 1 μL of 1% NP-40, and 1 μL of 50nM biotin-labeled DNA probes were incubated with 3 μg nuclear extract in a total volume of 20 μL at room temperature for 20 mins. Competition assays were performed with unlabeled DNA probes in concentrations exceeding the biotin-labeled probe by 200-fold. The DNA-protein complexes were resolved on 6% polyacrylamide gels and transferred to a nylon membrane (Invitrogen). Streptavidin-horseradish peroxidase was used to detect the complexes, which were then visualized on autoradiographic film (Genesee Scientific).

### QUANTIFICATION AND STATISTICAL ANALYSIS

The details of the statistical method used for identifying the differential gene expression using both DESeq2 and limma R packages are provided in the detailed methods above. Quantification of the immunostainings was performed by counting the positively stained cells in different fields of view. Statistical significance was determined using the Chi-square test using GraphPad Prism (version 9.0). The number of cells counted is indicated in the respective figure or its figure legend. For luciferase activity and qPCR analysis, data are represented as mean ± SD. Statistical significance was calculated by two-tailed unpaired Student’s t test using GraphPad Prism (version 9.0). The number of biological replications, data presentation, and statistical significance were indicated in respective figure legends.

## Supplementary Material

1

2

3

4

## Figures and Tables

**Table:** KEY RESOURCES TABLE

Reagent or resource	Source	Identifier
Antibodies		
Rabbit polyclonal anti-HA	Novus Biologicals	Cat#NB600-363; RRID: AB_10001504
Rabbit polyclonal anti-ETV4	Proteintech	Cat#10684-1-AP; RRID: AB_2100984
Rabbit polyclonal anti-DNMT3L	Abcam	Cat#ab194094; RRID: AB_2783649
Rabbit polyclonal anti-DNMT3L	A gift from Dr. Shinya Yamanaka (Kyoto University, Japan)	N/A
Rat monoclonal anti-GCNA1 (TRA98)	B-Bridge International	Cat# 73-003; RRID: AB_1056334
Guinea Pig polyclonal anti-IgG	Antibodies-Online	Cat# ABIN101961; RRID: AB_10775589
Goat anti-Rabbit IgG (H+L) Cross-Adsorbed Secondary Antibody, Alexa Fluor 488	Thermo Fisher Scientific	Cat# A-11008; RRID: AB_143165
Goat anti-Rat IgG (H+L) Secondary Antibody, Alexa Fluor® 555 conjugate	Thermo Fisher Scientific	Cat# A-21434; RRID: AB_141733
Donkey anti-Rabbit IgG (H+L) Highly Cross-Adsorbed Secondary Antibody, Alexa Fluor 647	Thermo Fisher Scientific	Cat# A-31573; RRID: AB_2536183
Donkey anti-Rat IgG (H+L) Highly Cross-Adsorbed Secondary Antibody, Alexa Fluor 594	Thermo Fisher Scientific	Cat# A-21209; RRID: AB_2535795
Bacterial and virus strains		
pGL3 vector	Promega	Cat# E1771
pRL-cmv vector	Promega	Cat# E2261
pLLU2G vector	Addgene	Cat#21620; RRID:Addgene_21620
pCAG-ERT2CreERT2	Addgene	Cat#13777; RRID:Addgene_13777
Biological samples		
Testis blocks	Mouse testes	N/A
Postnatal day (P) 0 – P2 testes	Mouse testes	N/A
Chemicals, peptides, and recombinant proteins		
Human, FGF-Basic, recombinant	Sigma-Aldrich	Cat# F0291-25UG
Collagenase type IV	Worthington Biochemical	Cat# LS004212
Deoxyribonuclease I	Worthington Biochemical	Cat# LS006355
(Z)-4-Hydroxytamoxifen	Sigma-Aldrich	Cat# H7904
4-Thiouridine	Millipore Sigma	Cat# T4509
Protein A-Tn5 transposase fusion protein	A gift from Dr. Steven Henikoff (Fred Hutchinson Cancer Research Center)	N/A
Critical commercial assays		
iScript cDNA synthesis Kit	BioRad	Cat# 170-8891
SsoAdvanceD Universal SYBR Green Supermix	BioRad	Cat# 172-5274
Dual Luciferase assay kit	Promega	Cat# E1960
QuantSeq 3′ mRNA-Seq Library Prep Kit	Lexogen	Cat# 015.24
NEBNext Ultra II Directional RNA Library Prep kit	New England Biolabs	Cat# E7765S
Deposited data		
RNaseq raw and analyzed data	Generated in this study	GEO: GSE165372
CUT&Tag raw and analyzed data	Generated in this study	GEO: GSE165372
iSLAMseq raw and analyzed data	Generated in this study	GEO: GSE165372
*Mus musculus*, release-96, Ensembl genome (GRCm38)	Ensembl genome	http://ftp.ensembl.org/pub/release-96/
*Mus musculus* (mm10) genome	UCSC	https://hgdownload.soe.ucsc.edu/downloads.html#mouse
Experimental models: Cell lines		
Mouse: GC1 cells	ATCC	CRL-2053
Human: HEK293T	ATCC	CRL-3216
Human: TCam-2 cells	de Jong et al., 2008	RRID: CVCL_T012
Experimental models: Organisms/strains		
Mouse: C57BL/6J	The Jackson Laboratory	JAX:000664
Mouse: B6.129-*Rhox10*^*tm1Wilk*^/J	The Jackson Laboratory	JAX: 035193
Mouse: B6;CBA-Tg(Pou5f1-EGFP)2Mnn/J	The Jackson Laboratory	JAX:004654
Oligonucleotides		
Oligonucleotides designed in this study	See Table S3 for oligonucleotide information	N/A
Software and algorithms		
Bowtie2 (v2.2.5)	Langmead and Salzberg, 2012	http://bowtie-bio.sourceforge.net/bowtie2/index.shtml
Samtools	Li et al., 2009	http://samtools.sourceforge.net/
Homer	Heinz et al., 2010	http://homer.ucsd.edu/homer/
IGV (version 2.6.2)	Robinson et al., 2011	https://software.broadinstitute.org/software/igv/
STAR (2.5.2b)	Dobin and Gingeras, 2015	https://github.com/alexdobin/STAR
SubRead	Liao et al., 2013	http://subread.sourceforge.net/
SLAM-DUNK (v0.4.3)	Neumann et al., 2019	https://github.com/t-neumann/slamdunk
R Version 4.0.0	https://www.r-project.org	N/A
DESeq2	Love et al., 2014	N/A
limma (v3.42.2)	Ritchie et al., 2015	N/A
GraphPad Prism (v9.0)	N/A	https://www.graphpad.com/scientific-software/prism/
The database for annotation, visualization and integrated discovery (DAVID) v6.8	Huang et al., 2009	https://david.ncifcrf.gov/

## Highlights

- RHOX10 directly targets many other transcription factors (TFs) in male germ cells
- Temporally distinct gene waves are regulated by RHOX10
- RHOX10 drives pro-spermatogonial differentiation via the TFs DMRT1 and ZBTB16
- The regulation of DMRT1 by RHOX family TFs is a conserved response
