# Supplementary material for: RHOX10 drives mouse spermatogonial stem cell establishment through a transcription factor signaling cascade
Source: Cell Rep. Author manuscript; Available in PMC 2021 Aug 11. (PMC8357189; doi:10.1016/j.celrep.2021.109423)
Supplement: 1 [file NIHMS1726847-supplement-1.pdf]

Cell Reports, Volume *36*

Supplemental information

RHOX10 drives mouse spermatogonial stem cell establishment through a transcription factor signaling cascade

Kun Tan, Hye-Won Song, and Miles F. Wilkinson

Cell Reports, Volume *36*

Supplemental information

RHOX10 drives mouse spermatogonial stem cell establishment through a transcription factor signaling cascade

Kun Tan, Hye-Won Song, and Miles F. Wilkinson

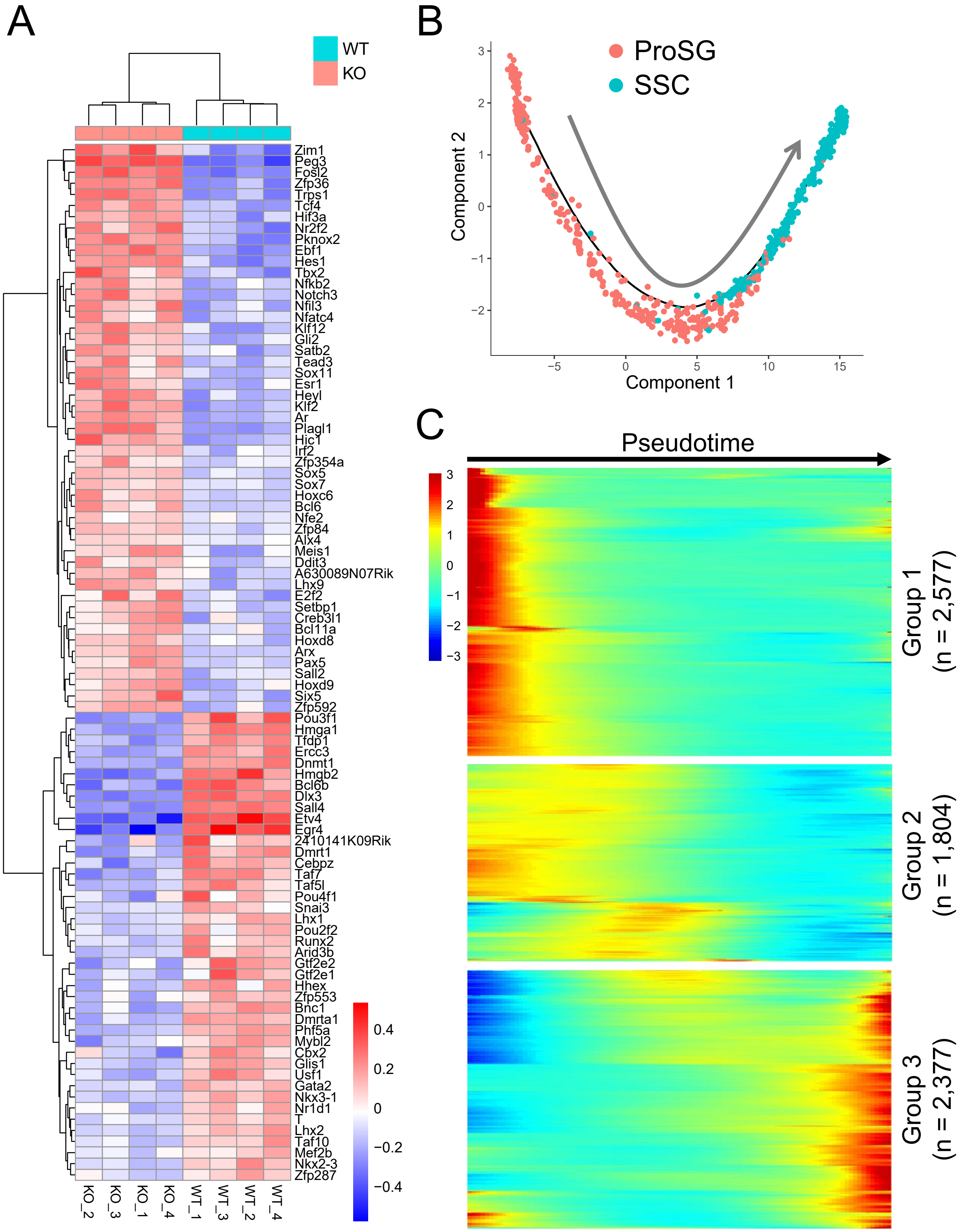

Figure S1. ***Rhox10*****-regulated genes in developing germ cells, Related to Figure 1.**
(A) Hierarchical clustering of TF genes exhibiting differential expression in the *Rhox10*-null vs. control germ cell samples described in Figure 1B (4 biological replicates from each genotype are shown).
(B) Monocle pseudotime trajectory analysis of the indicated cell clusters defined in Figure 1E.
(C) Heatmap depicting the expression pattern of genes in the 3 gene groups defined by pseudotime analysis in B. Each gene group has a unique expression pattern. Top: inferred developmental direction. Right: the number of DEGs in each group.

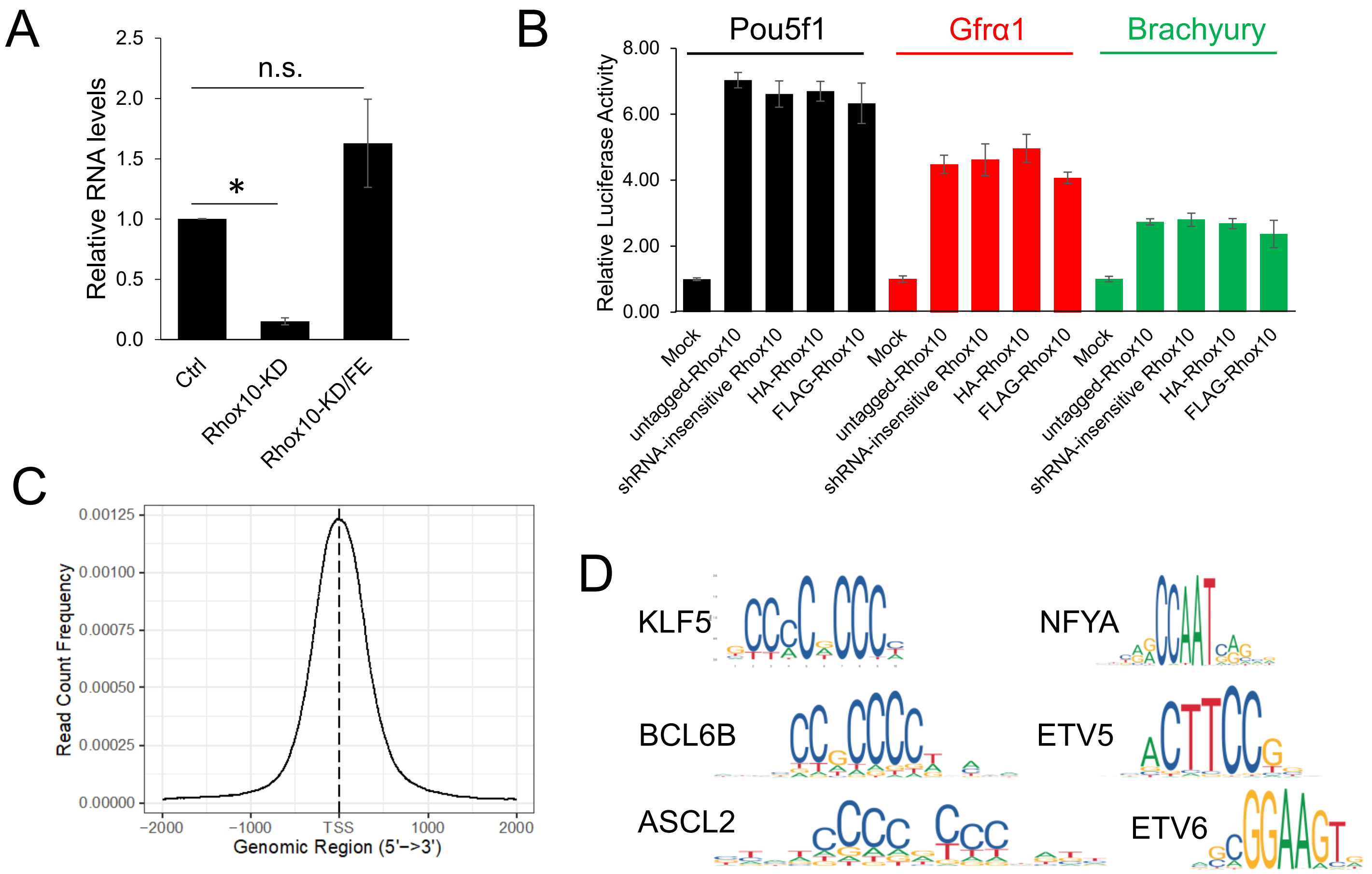

Figure S2. CUT&Tag analysis, Related to Figure 2.

(A) qPCR analysis of *Rhox10* expression in control (Ctrl) GS cells or in GS cells after shRNA-mediated *Rhox10* knock-down (KD) with or without forced expression (FE) of siRNA-resistant transduced *Rhox10*. Data are represented as mean ± SD (n = 3). Staistical significance was determined using the two-tailed unpaired Student's t test. *p < 0.05.

(B) Luciferase analysis of GC1 cells transiently transfected with constructs harboring promoters from the indicated genes ligated upstream of the Firefly luciferase gene. The cells were also co-transfected with the indicated *Rhox10* expression vectors and a *Rhox10* shRNA construct that depletes endogenous *Rhox10*. Data representation and statistical significance (n = 3) as in panel A.

(C) Distribution of RHOX10-occupancy peak reads ±2000 bp around the TSS of protein-coding genes, as determined by the CUT&Tag analysis described in Figure 2.

(D) TFs that bind sequence motifs similar those inferred for RHOX10 in this study. Of note, KLF5, BCL6B, and ASCL2 have motifs related to RHOX10 motif #1 in Figure 2C (the opposite strand is shown here); ETV6 has a motif related to RHOX10 motif #5 in Figure 2C (the opposite strand is shown here).

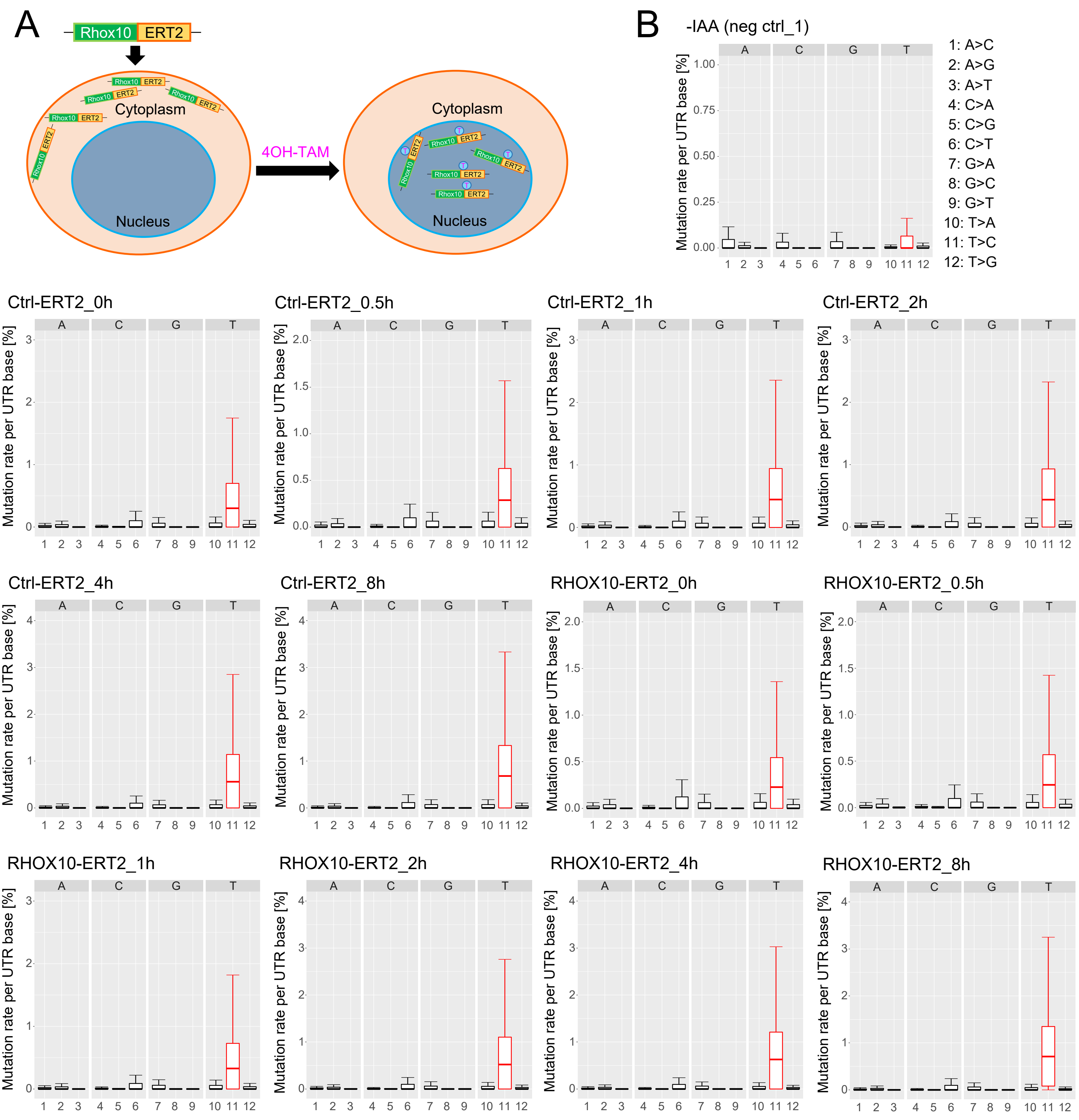

Figure S3. iSLAMseq analysis, Related to Figure 3.

(A) Schematic showing how rapid RHOX10 activation was achieved using the ERT2-TAM system.

(B) The conversion rates of the iSLAMseq samples (see Figure 3). Only one biological replicate from each time point is shown. The key is shown on the top right.

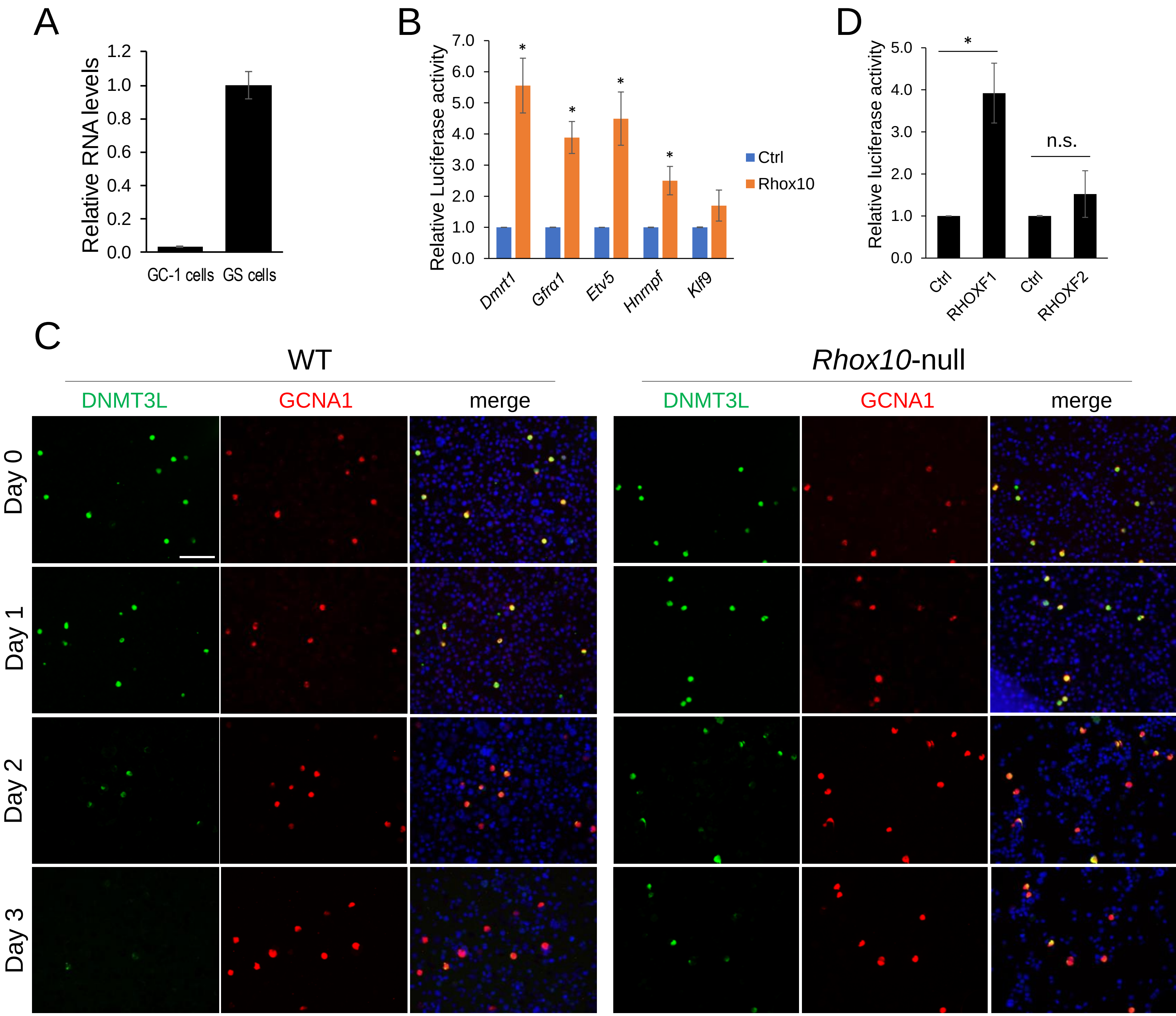

Figure S4. RHOX10 regulates the transcription of key SSC genes and drives ProSG differentiation in vitro, Related to Figure 4. (A) qPCR analysis of *Rhox10* expression in GS and GC1 cells, the latter of which largely lacks endogenous *Rhox10* expression (*Ct* value >32). Data are represented as mean ± SD. (n = 3). Statistical significance was determined using the Student's t-test. (B) Luciferase analysis of constructs harboring promoters from the indicated genes ligated upstream of the Firefly luciferase gene (see Figure 4A). These reporter constructs were transiently co-transfected with an *Rhox10*-expression vector into P19 cells. Data representation and statistical significance (n = 3) as in panel A. *p<0.05. (C) Immunofluorescence (IF) analysis of isolated P0 testicular cells of the indicated genotype cultured *in vitro*. Cells were co-stained with antisera against DNMT3L and GCNA1. Cell nuclei were stained with DAPI (blue). Scale bar, 75 µm. (D) Luciferase analysis of a construct harboring the human *DMRT1* promoter ligated upstream of the Firefly luciferase gene. This construct was transiently co-transfected into HEK293T cells with the *RHOXF1 or RHOXF2* expression vectors, as indicated. Data representation and statistical significance (n = 3) as in panel A. *p<0.05.
